# Discovery and optimization of a broadly-neutralizing human monoclonal antibody against long-chain α-neurotoxins from snakes

**DOI:** 10.1038/s41467-023-36393-4

**Published:** 2023-02-08

**Authors:** Line Ledsgaard, Jack Wade, Timothy P. Jenkins, Kim Boddum, Irina Oganesyan, Julian A. Harrison, Pedro Villar, Rachael A. Leah, Renato Zenobi, Sanne Schoffelen, Bjørn Voldborg, Anne Ljungars, John McCafferty, Bruno Lomonte, José M. Gutiérrez, Andreas H. Laustsen, Aneesh Karatt-Vellatt

**Affiliations:** 1 Department of Biotechnology and Biomedicine, Technical University of Denmark, DK-2800 Kongens Lyngby, Denmark; 2 Sophion Bioscience, DK-2750 Ballerup, Denmark; 3 Department of Chemistry and Applied Biosciences, ETH Zurich, CH-8093 Zurich, Switzerland; 4 IONTAS Ltd., Cambridgeshire, CB22 3FT UK; 5 Instituto Clodomiro Picado, Facultad de Microbiología, Universidad de Costa Rica, San José, 11501-2060 Costa Rica

**Keywords:** Applied immunology, Molecular biology, Biological techniques, Antibody therapy, Synthetic biology

## Abstract

Snakebite envenoming continues to claim many lives across the globe, necessitating the development of improved therapies. To this end, broadly-neutralizing human monoclonal antibodies may possess advantages over current plasma-derived antivenoms by offering superior safety and high neutralization capacity. Here, we report the establishment of a pipeline based on phage display technology for the discovery and optimization of high affinity broadly-neutralizing human monoclonal antibodies. This approach yielded a recombinant human antibody with superior broadly-neutralizing capacities in vitro and in vivo against different long-chain α-neurotoxins from elapid snakes. This antibody prevents lethality induced by *Naja kaouthia* whole venom at an unprecedented low molar ratio of one antibody per toxin and prolongs the survival of mice injected with *Dendroaspis polylepis* or *Ophiophagus hannah* whole venoms.

## Abstract (web summary)

The treatment of snakebite envenoming is currently suboptimal. Existing antivenoms often lack efficacy and may cause adverse reactions. Here, the authors derive, develop, and demonstrate the utility of toxin-specific broadly-neutralizing human monoclonal antibodies with established reactivity across related venom toxins from different snake species and show efficacy in rodent models.

## Introduction

Each year, snakebite envenoming exacts a high death toll and leaves hundreds of thousands of other victims maimed for life^1^. Antivenoms based on polyclonal antibodies isolated from the plasma of immunized animals are currently the only specific treatment option against severe envenomings^2,3^. While these medicines are essential and life-saving and will remain a cornerstone in snakebite therapy for years to come, an opportunity now exists to modernize treatment by exploiting the benefits of recombinant DNA and antibody technology^4^. Indeed, recombinant antibodies and antibody fragments have already been generated against a variety of snake venom toxins^5–8^, as well as multiple studies involving monoclonal antibodies derived using hybridoma technology have been reported^9–12^ (see Laustsen et al.^13^ for a comprehensive overview and Pucca et al.^2^ for an overview of the historical context). Within this area of research, it has also been demonstrated that monoclonal antibodies targeting snake venom toxins can be developed using various platforms, such as phage display technology^6^, an in vitro methodology that can be used to actively select for antibodies with high-affinity and cross-reactivity^14,15^. In addition, the use of human antibody libraries in combination with phage display technology allows for the discovery of fully human antibodies that are likely to have high treatment tolerability in patients^16^.

It has been speculated that monoclonal antibodies developed by these means could be used to formulate recombinant antivenoms that elicit fewer adverse reactions, are cost-competitive to existing therapy, and can be fine-tuned to have superior efficacy^16–20^. Phage display technology could be particularly valuable for discovering monoclonal antibodies against highly potent toxins with low immunogenicity that fail to elicit a strong antibody response in animals used for immunization^21,22^. This is the case for low molecular mass neurotoxins and cytotoxins of the three-finger toxin (3FTx) family, which are abundant in Elapidae venoms, such as cobra and mamba venoms^23–26^. These elapid venoms, unlike Viperidae venoms, typically consist of neurotoxic and cytoxic components that elicit tissue damage as well as paralysis in bite victims^1^. However, antibodies derived directly from naïve libraries often lack sufficiently high affinity to enable toxin neutralization^15^. Affinity can be improved by further site-directed or random mutagenesis of the antibody paratopes, which can also lead to broadening of the neutralizing capacity of naïve antibodies^27^. However, in addition to mutation of the antibody binding regions, retaining the heavy chains and exploring alternative light chains, a technique known as light chain-shuffling, has shown significant promise as well^21,28^. Here, a phage display library is generated by pairing a heavy or light chain from a specific antibody with a naïve repertoire of the partner chain and performing a new selection campaign^15^. Nevertheless, until now it remained unknown whether this technology could be used to generate antibodies that possess high affinity while simultaneously having a broad neutralization capacity, i.e., are able to neutralize several related toxins from the venoms of different snake species.

Previously, using a naïve human scFv-based phage display library^15^, we described the discovery and characterization of the human monoclonal antibody, 368_01_C05, against α-cobratoxin (P01391), a potent neurotoxin from the monocled cobra, *Naja kaouthia*. Notably, this antibody could prolong the survival of mice injected with lethal doses of α-cobratoxin, although it failed to prevent lethality^15^. As a follow-up development, in the present study we constructed light-chain-shuffled antibody libraries based on this clone with the aim of using a phage display-based cross-panning campaign to simultaneously improve the affinity and expand the neutralizing capacity of the antibody against α-neurotoxins from the venoms of several snake species. Cross-panning was carried out between α-cobratoxin^29^ and α-elapitoxin^30^, a neurotoxin from the venom of the black mamba, *Dendroaspis polylepis*^25^. These two α-neurotoxins share 70% sequence identity and both cause neuromuscular blockade by binding to the nicotinic acetylcholine receptor (nAChR) in muscle cells^29,30^.

In this work, we “cross-panned” the chain-shuffled scFv library on these two toxins under stringent conditions to discover antibodies with improved affinity and cross-reactivity in comparison to the parent antibody. Using this strategy, we were able to generate an antibody that not only has improved affinity to α-cobratoxin, but also significantly broadened cross-neutralization capacity against other α-neurotoxins from the venoms of elapid snakes from the genera *Dendroaspis, Ophiophagus, Bungarus*, and *Naja*.

## Results

### Affinity maturation, cross-panning, and scFv characterization

**Fig. 1:**
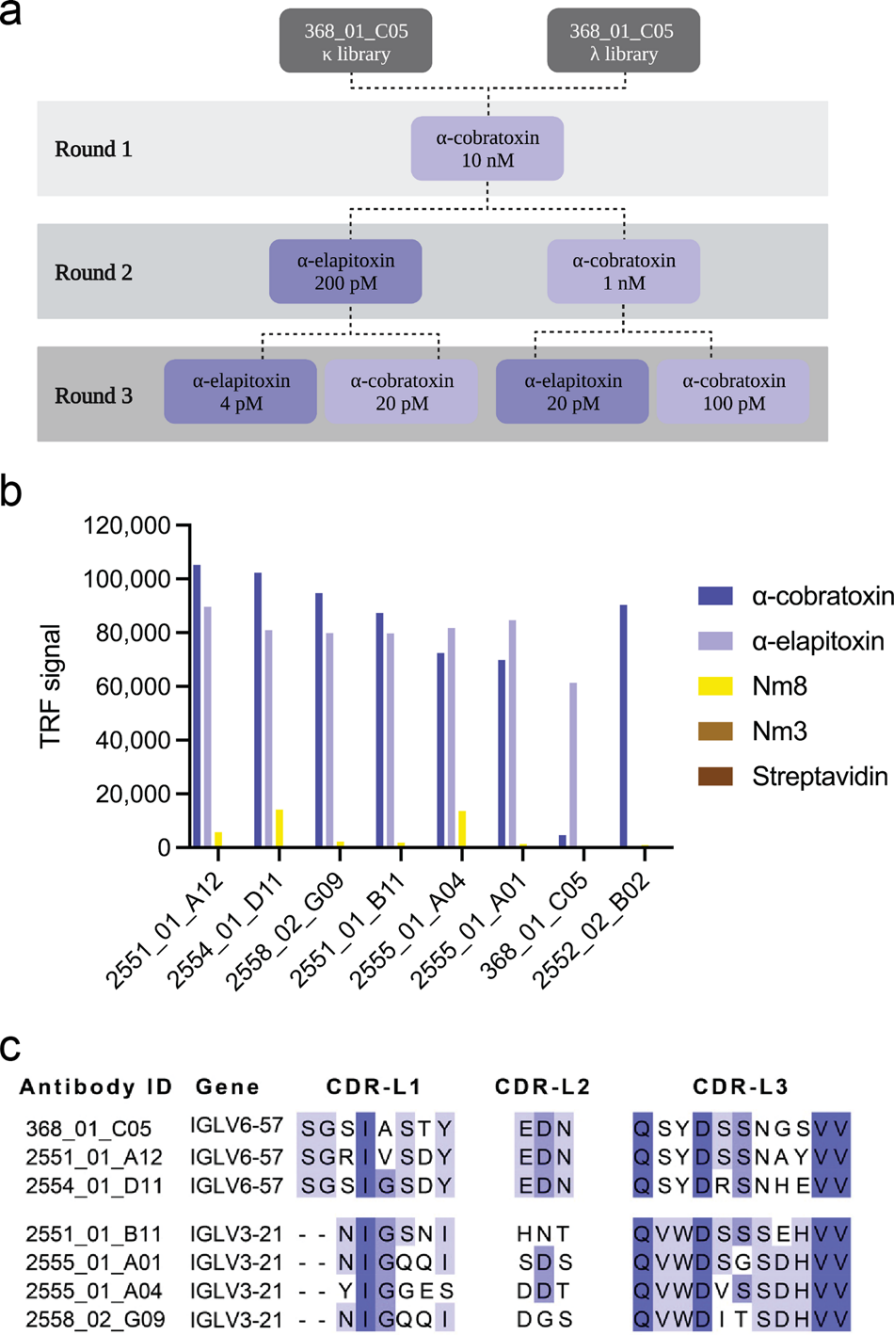
Cross-panning selection strategy as well as assay and sequence data for selected IgGs. **a** Selection strategy illustrating how cross-panning was performed, including antigen concentrations. **b** ENC DELFIA showing cross-reactivity of the top six-affinity matured IgGs (2551_01_A12, 2554_01_D11, 2558_02_G09, 2551_01_B11, 2555_01_A04, and 2555_01_A01) in comparison with parental IgG (368_01_C05) and clone 2552_02_B02 from a previously published study^15^. **c** Comparison of CDR-L1, CDR-L2, and CDR-L3 sequences for the top six chain-shuffled antibodies and the parental antibody.

Human light-chain-shuffled scFv-based phage display libraries were created by pairing the heavy chain of antibody 368_01_C05 with a naïve repertoire of human light chains. Then, phage display cross-pannings using two toxins with 70% sequence identity, α-cobratoxin from *N. kaouthia* and α-elapitoxin from *D. polylepis*, were conducted according to the overview provided in Fig. 1a. Phage display selection outputs from the third round were subcloned into the pSANG10-3F expression vector, and 736 monoclonal scFvs were tested for their ability to bind to α-cobratoxin, α-elapitoxin, and streptavidin in both direct dissociation-enhanced lanthanide fluorescence immunoassays (DELFIAs) and expression-normalized capture (ENC) DELFIAs. From here, 203 scFvs (all displaying binding to at least one of the two toxins with negligible binding to streptavidin) were randomly selected for sequencing. Of these, 67 scFvs were unique according to the sequence of their light chain CDR3 region, 2 of them having kappa light chains and the remaining 65 having lambda light chains. The top 62 clones, based on sequence diversity and binding behavior, were reformatted to the fully human IgG1 format. Following expression in HEK293 cells, ENC DELFIAs were run using the crude expression supernatant to rank the IgG binding to α-cobratoxin, α-elapitoxin, a venom fraction from *N. melanoleuca* (Nm8) containing a long neurotoxin homologous to OH-55 (Q53B58) and long neurotoxin 2 (P01388)^31^. In addition, streptavidin was included as negative control. This revealed that more than half of the clones were cross-reactive against all three toxins/venom fractions, demonstrating significant improvement in both binding and cross-reactivity when compared to the parental antibody.

To help guide the selection of lead candidates, the suitability of the 62 clones for future antibody development was investigated by characterizing biophysical properties that are indicative of their “developability” profiles. To this end, we analyzed the purity and nonspecific column interaction pattern of all IgGs using size-exclusion chromatography (SEC). In addition, the propensity for self-aggregation was evaluated in an AC-SINS assay, in which IgGs are concentrated around gold nanoparticles pre-coated with anti-Fc antibodies, where a reduced inter-particle distance (measured by increased plasmon wavelengths) indicates that the immobilized IgGs interact unspecifically^32^. For this analysis, we also included an IgG from a previous study (2552_02_B02)^15^, that has been reported to neutralize lethality induced by *N. kaouthia* whole venom in vivo, but had never been characterized for cross-reactivity to other long-chain α-neurotoxins nor been analyzed for its “developability” properties. The SEC data (% monomeric content and relative elution volumes—a metric for assessing nonspecific interaction with the SEC column), AC-SINS shifts, binding data, expression yields (full dataset see Table S1), and light chain germline diversity were used to select the top six antibody candidates for further characterization. These antibodies were named 2551_01_A12, 2554_01_D11, 2558_02_G09, 2551_01_B11, 2555_01_A04, and 2555_01_A01 (Fig. 1b and Table 1). Additionally, the data showed that the previously published IgG 2552_02_B02 had a poor developability score, both judged by its late elution in the SEC analysis and its high shift in the AC-SINS assay. In fact, this antibody performed at a similarly poor level as the ‘poor developability’ control (bococizumab, AC-SINC shift of 33 nm) that was used for comparison, whereas all antibodies derived from the 368_01_C05 parental clone possessed developability profiles similar to the ‘good developability’ control antibody (Aliricumab, AC-SINS shift of 3 nm). In addition, 2552_02_B02 showed no cross-reactivity to any of the long-chain α-neurotoxins it was tested against, clearly distinguishing its binding profile from the antibodies derived from the 368_01_C05 parent (Fig. 1b).

**Table 1:** AC-SINS shift, SEC analysis results (% monomer content and relative elution volume), and transient expression yields for the top six light chain-shuffled IgGs (2551_01_A12, 2554_01_D11, 2558_02_G09, 2551_01_B11, 2555_01_A04, and 2555_01_A01) in comparison with the parental IgG (368_01_C05)

	AC-SINS	SEC analysis		Production
Antibody ID	Shift (nm)	Monomer (%)	Elution (mL)	Yield (mg/L)
2551_01_A12	3	100.0	1.48	18.1
2554_01_D11	1	94.8	1.48	47.1
2558_02_G09	1	95.6	1.48	38.5
2551_01_B11	1	96.2	1.50	30.4
2555_01_A04	1	100.0	1.47	25.8
2555_01_A01	1	96.7	1.47	45.4
368_01_C05	0	97.5	1.45	30.0
2552_02_B02*	32	100.0	1.82	22.6

*IgG 2552_02_B02 from a previous study was also included.

Analysis of the antibody sequences revealed that the six affinity matured antibodies had light chains belonging to two different germlines, germline IGLV3-21 for 2551_01_B11, 2555_01_A01, 2555_01_A04, and 2558_02_G09 and germline IGLV6-57 for 2551_01_A12 and 2554_01_D11. The parental antibody had germline IGLV6-57, meaning that two of the six affinity matured antibodies had light chains belonging to the same germline as the parental antibody. From the comparison of the three light chain CDR regions of the antibodies presented in Fig. 1c, it could also be seen that for the two antibodies maintaining the parental germline, the CDR-L2 was identical to the parental, whereas the CDR-L1 and CDR-L3 had 2–3 amino acid differences. For the remaining four antibodies with different light chain germline, all V_L_ CDR sequences were significantly different from the parent antibody sequence.

To evaluate whether the light-chain-shuffling campaign generated antibodies with improved affinity, surface plasmon resonance (SPR) was used to determine the affinity of the top six antibodies as well as the parental antibody. To this end, all antibodies were reformatted to the monovalent Fab format to measure the 1:1 binding kinetics of each antibody against both α-cobratoxin and α-elapitoxin (for SPR sensorgrams see Fig. S1). The data showed that all six antibodies displayed higher affinity for both toxins than the parental antibody (Table 2). The largest improvement was observed for 2551_01_A12 and 2554_01_D11 (32 and 50-fold improvement of binding to α-cobratoxin and 13 and 8-fold improvement of binding to α-elapitoxin, respectively), providing both antibodies with low single-digit nanomolar affinities to both toxins. Thus, a significant improvement in both affinity and cross-reactivity was observed. Antibodies 2551_01_A12 and 2554_01_D11 were selected for further characterization based on affinity, cross-reactivity, expression yield, and developability data.

**Table 2:** Affinity measurements between antibodies and toxins using Surface Plasmon Resonance (SPR)

	α-cobratoxin			α-elapitoxin		
Antibody ID	*K*_D_ (nM)	*k*_on_ (M·s)	*k*_off_ (s^−1^)	*K*_D_ (nM)	*k*_on_ (M·s)	*k*_off_ (s^−1^)
2551_01_A12	2.79	1.80 × 10^5^	5.02 × 10^−4^	1.12	1.25 × 10^5^	1.40 × 10^−4^
2554_01_D11	1.78	1.28 × 10^5^	2.29 × 10^−4^	1.69	1.05 × 10^5^	1.77 × 10^−4^
2558_02_G09	2.77	2.14 × 10^5^	5.94 × 10^−4^	3.04	1.39 × 10^5^	4.22 × 10^−4^
2551_01_B11	4.27	1.17 × 10^5^	5.00 × 10^−4^	2.87	6.37 × 10^5^	1.83 × 10^−4^
2555_01_A04	7.46	2.22 × 10^5^	1.65 × 10^−3^	2.21	9.26 × 10^5^	2.05 × 10^−4^
2555_01_A01	8.41	2.02 × 10^5^	1.70 × 10^−3^	1.81	1.00 × 10^5^	1.81 × 10^−4^
368_01_C05	89.6	1.83 × 10^4^	1.64 × 10^−2^	14.3	6.47 × 10^4^	9.25 × 10^−4^

SPR was used to measure the affinity of the top six chain-shuffled antibodies (2551_01_A12, 2554_01_D11, 2558_02_G09, 2551_01_B11, 2555_01_A04, and 2555_01_A01) and the parental antibody (368_01_C05) in the Fab format to both α-cobratoxin and α-elapitoxin. The dissociation constants, on-rates, and off-rates are provided. For sensorgrams, see Fig. S1.

Because the binding profiles of the cross-reactive antibodies derived from antibody 368_01_C05 were significantly different from antibody 2552_02_B02, SPR was used to determine if the antibodies bound the same or overlapping epitopes on α-cobratoxin (see Fig. S2). Using 2554_01_D11 as a representative of the cross-reactive antibodies, this study revealed that neither of the two antibodies 2552_02_B02 or 2554_01_D11 could bind α-cobratoxin if the other antibody was already bound to the toxin, suggesting that the antibodies recognized the same or overlapping epitopes.

### Native mass spectrometry reveals cross-reactivity to several toxins from elapid snakes of three different genera

To further explore the cross-reactivity of the discovered antibodies, IgG 2554_01_D11 was tested for its binding to toxins in five whole venoms, including *N. kaouthia*, *N. melanoleuca*, *N. naja*, *Ophiophagus hannah*, and *D. polylepsis*. These venoms from African (*D. polylepsis* and *N. melanoleuca)* and Asian (*N. kaouthia*, *N. naja* and *O. hannah)* snakes all possess a relatively high content of long-chain α-neurotoxins, ranging from 13.2% for *D. polylepsis*^25^ to 55% for *N. kaouthia*^24^, except *N. naja*, which has been reported to have a long-chain α-neurotoxin content of approximately 2–5%^33^. For this purpose, native mass spectrometry (MS), was used to investigate the interactions between the antibody and toxins from the four snake venoms.

**Fig. 2:**
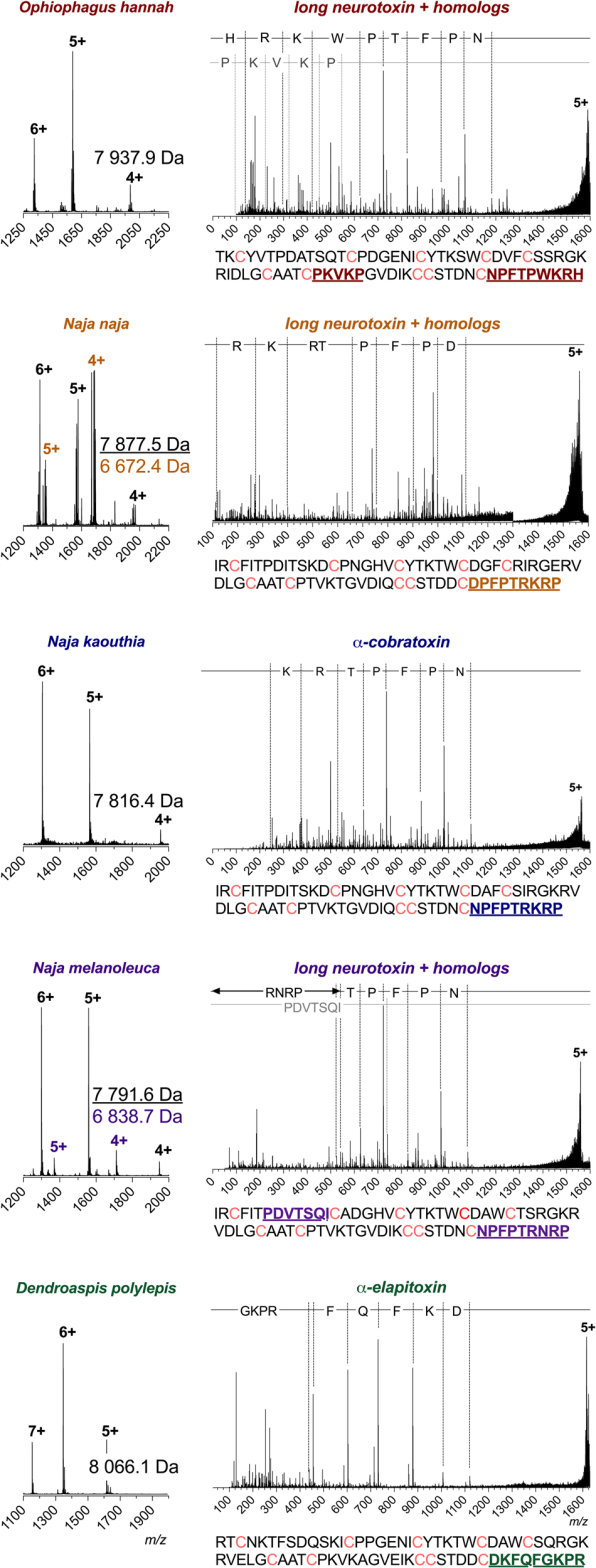
Intact masses and top-down sequence analysis of toxins bound by 2554_01_D11. Names above the mass spectra have been color-coded for each species as follows: *O. hannah* (red), *N. naja* (orange), *N. kaouthia* (blue), *N. melanoleuca* (purple) and *D. polylepis* (green). The spectra on the left-hand side show the charge state distribution for the toxins ejected from the antibody complex by applying a high cone voltage, where the masses of the identified toxins are given in Daltons. The top-down sequence spectra for the most prominent charge state of each toxin are shown on the right-hand side. The difference in *m/z* is outlined via dotted lines on top and matches the specific amino acid or peptide. The full amino acid sequence for the proposed identity of the toxins is given below each spectrum, with the matching peptides found during the top-down analysis colored and underlined. Cysteines in the sequence are colored pink.

Prior to native mass spectrometry analysis, the venoms and IgG were fractionated using SEC (Fig. S3). IgG was mixed with each of the SEC-generated toxin fractions before analysis using native MS to determine binding (Fig. S3). This analysis revealed that 2554_01_D11 only bound toxins of masses in the range expected for the group of three-finger toxins (3FTx) to which all α-neurotoxins belong. To identify the toxins, the toxin:antibody complexes were isolated using MS/MS and subjected to collisional dissociation to eject the toxins from the antibody, allowing their intact mass to be determined. The primary dissociation products from these experiments were proteins of masses between 7800 and 8200 Da, corresponding to typical masses of long-chain α-neurotoxins (Fig. 2).

The sequences of the toxins bound by 2554_01_D11 were investigated via top-down proteomics to confirm the identities of these toxins. For these experiments, the toxin:antibody complexes were purified using SEC. Toxins were dissociated from the antibody by applying a high cone voltage. This is a focusing voltage applied to the cone, which is located in the source region of the instrument. Increasing this voltage leads to harsh conditions that can dissociate noncovalent complexes. Since this dissociation occurs before the quadrupole, the most prominent charge state of each ejected toxin could then be isolated using MS/MS for top-down sequencing. This isolation is important, as it ensures that the peptide fragmentation peaks only correspond to the toxin of interest. For the toxins with masses between 7800 and 8200 Da, only one readily discernible peptide fragment series was detected for each precursor ion. The limited amount of sequence data obtained from these experiments is attributed to the presence of disulfide bonds present in snake venom toxins, which cannot be broken using this fragmentation technique^34–36^.

A BLAST search against all available elapid protein sequences revealed that the peptide sequences obtained by top-down analysis were unique to long-chain α-neurotoxins and that each peptide only had one complete match to long-chain α-neurotoxin homologs from the investigated venom. Sequence data combined with the detected masses of the toxins revealed that 2554_01_D11 was capable of binding to long-chain α-neurotoxin-containing SEC fractions across all tested venoms. This suggested that this antibody is cross-reactive against long-chain α-neurotoxins present in all five tested venoms, further highlighting the broadly cross-reactive behavior of 2554_01_D11.

**Fig. 3:**
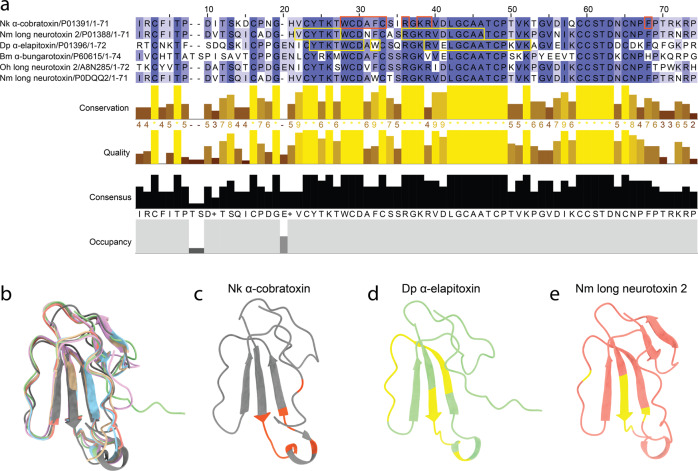
Alignment and epitope identification of all investigated long-chain α-neurotoxins, i.e., α-cobratoxin (P01391/1CTX) from *N. kaouthia*, α-elapitoxin (P01396/AF-P01396) from *D. polylepis*, α-bungarotoxin (P60615/1HC9) from *B. multicinctus*, long neurotoxin 2 (A8N285/AF-A8N285) from *O. hannah*, and long neurotoxin (P0DQQ2), long neurotoxin 4 from *N. naja* (P25672/AF-P25672), and long neurotoxin 2 (P01388/AF-P01388) from *N. melanoleuca*. **a** Sequence alignment using Clustal Omega with boxes indicating residues involved in binding to the nicotinic acetylcholine receptor (orange) or bound by antivenom antibodies (yellow). **b** Structural alignment in ChimeraX with the following colors representing each toxin: orange (long neurotoxin 2 from *N. melanoleuca*), beige (long neurotoxin 2 from *O. hannah*), purple (α-bungarotoxin), green (α-elapitoxin from *D. polylepis*), blue (long neurotoxin 4 from *N. naja*), and gray (α-cobratoxin from *N. kaouthia*). **c** Amino acid residues on α-cobratoxin known to be involved in binding to its native target, *i.e*., the nicotinic acetylcholine receptor^39^ (orange). **d** Amino acid residues in α-elapitoxin suggested to be bound by antivenom antibodies based on high-density peptide microarray analysis^37^ (yellow). **e** Amino acid residues in long neurotoxin 2 from *N. melanoleuca* suggested to be bound by antivenom antibodies based on high-density peptide microarray analysis^37^ (yellow).

The toxin homologs specifically identified to be bound by 2554_01_D11 were long neurotoxin 2 (A8N285) from *O. hannah*, α-cobratoxin (P01391) from *N. kaouthia*, long neurotoxin 2 (P01388) and long neurotoxin (P0DQQ2) from *N. melanoleuca*, long neurotoxin 4 (P25672) from *N. naja*, and α-elapitoxin (P01396) from *D. polylepis*. In addition, the antibody was shown to bind α-bungarotoxin (P60615) from *B. multicinctus* using SPR (Fig. S4). The average sequence similarity of the seven toxins was 62% (stdev: 9.9%), with an identity of 38% across all toxins; a total of 28 amino acid positions (primarily located at the active site) were identical across all toxins (Fig. 3a). The highest identity was observed between α-cobratoxin and long neurotoxin 2 from *N. melanoleuca* (83%), and the lowest identity was observed between long neurotoxin 2 from *N. melanoleuca* and α-bungarotoxin (51%). Additionally, a structural comparison was performed via root-mean-square deviation (RMSD) and revealed a mean pruned/total similarity of 0.81 Å/3.1 Å (stdev: 0.26 Å/1.23 Å), respectively; the best match appeared to be between long neurotoxin and long neurotoxin 2 from *N. melanoleuca* (0.23 Å/0.23 Å) and the poorest match appeared to be between long neurotoxin 2 from *O. hannah* and α-cobratoxin (pruned: 1.18 Å) and α-elapitoxin and α-bungarotoxin (total: 4.5 Å; Fig. 3b). For α-cobratoxin, the amino acid residues involved in binding to the nicotinic acetylcholine receptor have been highlighted both in the sequence (Fig. 3a) and in the structure on the toxin (Fig. 3c). Additionally, the residues that, through a high-density peptide microarray-based study^37^, have been identified to be involved in the binding between antivenom-derived antibodies and α-elapitoxin and long neurotoxin 2 from *N. melanoleuca*, have been highlighted in the toxin sequence in Fig. 3a and in the toxin structure in Figs. 3d, e.

### Increased in vitro neutralization potency and broadening of cross-neutralization

**Fig. 4:**
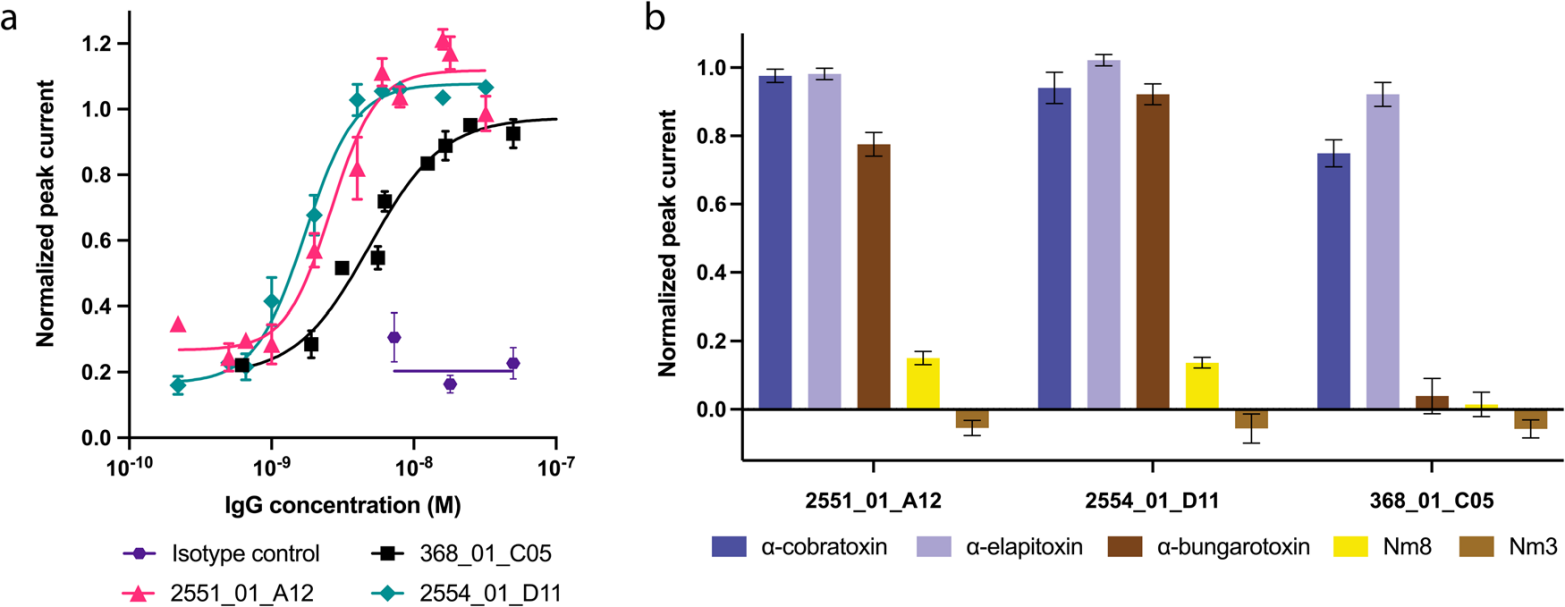
Electrophysiological determination of the in vitro cross-neutralizing potential of 2551_01_A12, 2554_01_D11, and 368_01_C05. Automated patch-clamp experiments were performed to determine the ability of the antibodies to prevent the current-inhibiting effect that α-neurotoxins exert on the nAChR. **a** Concentration-response curves illustrating how increasing concentrations of the three antibodies prevent nAChR blocking by α-cobratoxin (SD shown, each datapoint based on at least *n* = 4 independent experiments, one experiment equals 10 cells). **b** Single concentration plot outlining the cross-neutralizing potential of the antibodies against α-cobratoxin from *N. kaouthia*, α-elapitoxin from *D. polylepis*, α-bungarotoxin from *B. multicinctus*, and Nm8 from *N. melanoleuca* (SD shown, *n* = 6 independent experiments, of which each equals 10 cells). In addition, a negative control Nm3, a fraction from *N. melanoleuca* venom containing a short α-neurotoxin, was included. The toxin to antibody molar ratios used were 1:22 for α-cobratoxin, 1:40 for α-elapitoxin, 1:5 for α-bungarotoxin, 1:2.3 for Nm8, and 1:3.2 for Nm3.

After having established the broadly cross-reactive nature of one of the top two chain-shuffled antibodies (2554_01_D11), automated patch-clamp technology was applied to assess whether binding translated into functional neutralization in vitro for 2551_01_A12 and 2554_01_D11, as well as for the parental clone, 368_01_C05. Here, a human derived cell line endogenously expressing nAChR was used to measure the acetylcholine-dependent current. α-cobratoxin inhibited this current in a concentration-dependent manner, and the IC_80_ value for the toxin was determined. Thereafter, the concentration-dependent neutralization of the current-inhibiting effect of α-cobratoxin by the three antibodies was determined. The results demonstrated that all three antibodies were able to fully neutralize the effects of α-cobratoxin, whereas an irrelevant isotype control antibody (recognizing a dendrotoxin) had no effect (Fig. 4a). The parental antibody, 368_01_C05 neutralized α-cobratoxin-mediated inhibition of acetylcholine-dependent currents with an EC_50_ value of 4.9 nM and a relatively shallow concentration-response curve slope. In contrast, the optimized antibodies, 2551_01_A12 and 2554_01_D11 exhibited improved EC_50_ values of 2.6 and 1.7 nM, respectively, with steeper slopes for the concentration-response curves. These EC_50_ values translate into toxin:antibody molar ratios of 1:1.23 for 368_01_C05, 1:0.65 for 2551_01_A12, and 1:0.43 for 2554_01_D11. Since each IgG has two binding sites, the theoretically lowest amount of IgG needed to neutralize the effect of one toxin would be 0.5 IgGs.

To determine if the increased cross-reactivity to other α-neurotoxins translated into cross-neutralization, a single concentration antibody screen was set up using the Qube 384 system. Here, the three antibodies (368_01_C05, 2551_01_A12, and 2554_01_D11) were tested against α-cobratoxin from *N. kaouthia*, α-elapitoxin from *D. polylepis*, and Nm8 from *N. melanoleuca*, which all were toxins that 2554_01_D11 had been shown to bind through native MS. In addition, α-bungarotoxin was included, as it has 58% sequence identity to α-cobratoxin, is commercially available, and is an important toxin to neutralize in the venom of *B. multicinctus*. As a control, Nm3, a venom fraction from *N. melanoleuca* containing a short-chain α-neurotoxin that also binds to the nAChR but is not bound by any of the three antibodies, was included. This automated patch-clamp screening revealed that α-cobratoxin and α-elapitoxin could be neutralized by all three antibodies in this assay (Fig. 4b). Additionally, the chain-shuffled clones were able to neutralize α-bungarotoxin and partially neutralize the α-neurotoxins present Nm8, none of which was achieved by the parental clone. Collectively, the results of the in vitro neutralization assays using automated patch-clamp demonstrated that the chain-shuffled antibodies were both more potent in their neutralization of α-cobratoxin, as well as more broadly neutralizing than the parental antibody, inhibiting the effect of α-neurotoxins from snakes of three different genera inhabiting both Asia and Africa. Based on binding, developability, affinity, expression, and in vitro neutralization data, 2554_01_D11 was selected as the top candidate for in vivo testing.

### In vitro neutralization data translate to complete or partial in vivo neutralization of snake venoms from different genera and continents

**Fig. 5:**
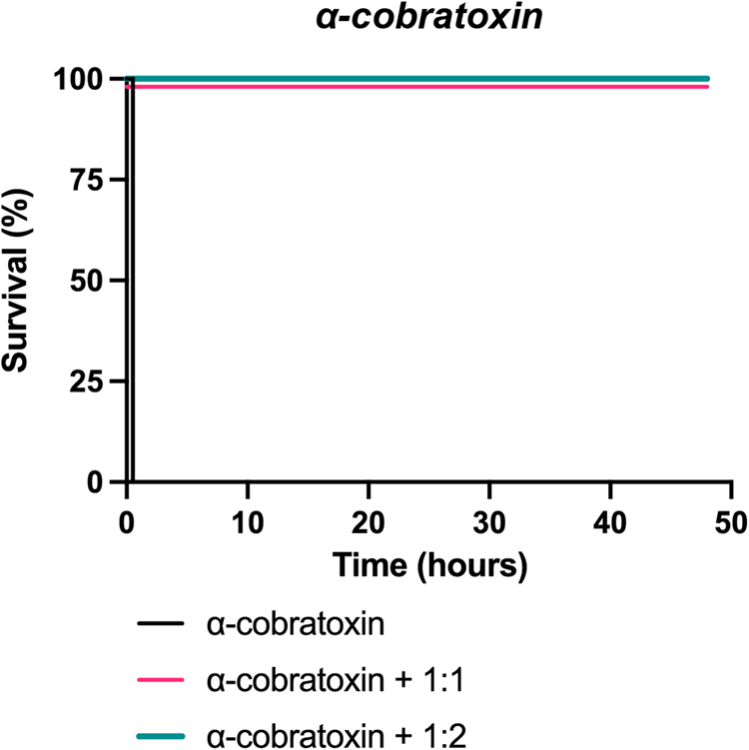
Kaplan–Meier survival curves for mice challenged with α-cobratoxin preincubated with or without the antibody 2554_01_D11. Two LD50s of α-cobratoxin (*N. kaouthia*) were preincubated with the antibody, 2554_01_D11, at various neurotoxin:antibody ratios and then administered i.v. into groups of four mice. Controls included mice receiving α-cobratoxin alone (see Materials and Methods for details). Signs of toxicity were observed, and deaths were recorded for a maximum period of 48 h.

To verify that the in vitro cross-neutralization potential of 2554_01_D11 translated into in vivo cross-neutralization, animal experiments were set up to evaluate the ability of the antibody to prevent or delay venom-induced lethality. First, we evaluated the neutralization of α-cobratoxin. Two LD_50_s of this neurotoxin were incubated with 2554_01_D11 in a 1:1 or 1:2 toxin:antibody molar ratio, and the toxicity was tested i.v. in mice. Animals receiving the toxin alone died within 30 min of injection with evident signs of neurotoxic paralysis, whereas all mice receiving the toxin incubated with the antibody, at the two molar ratios tested, survived without showing signs of intoxication (Fig. 5).

**Fig. 6:**
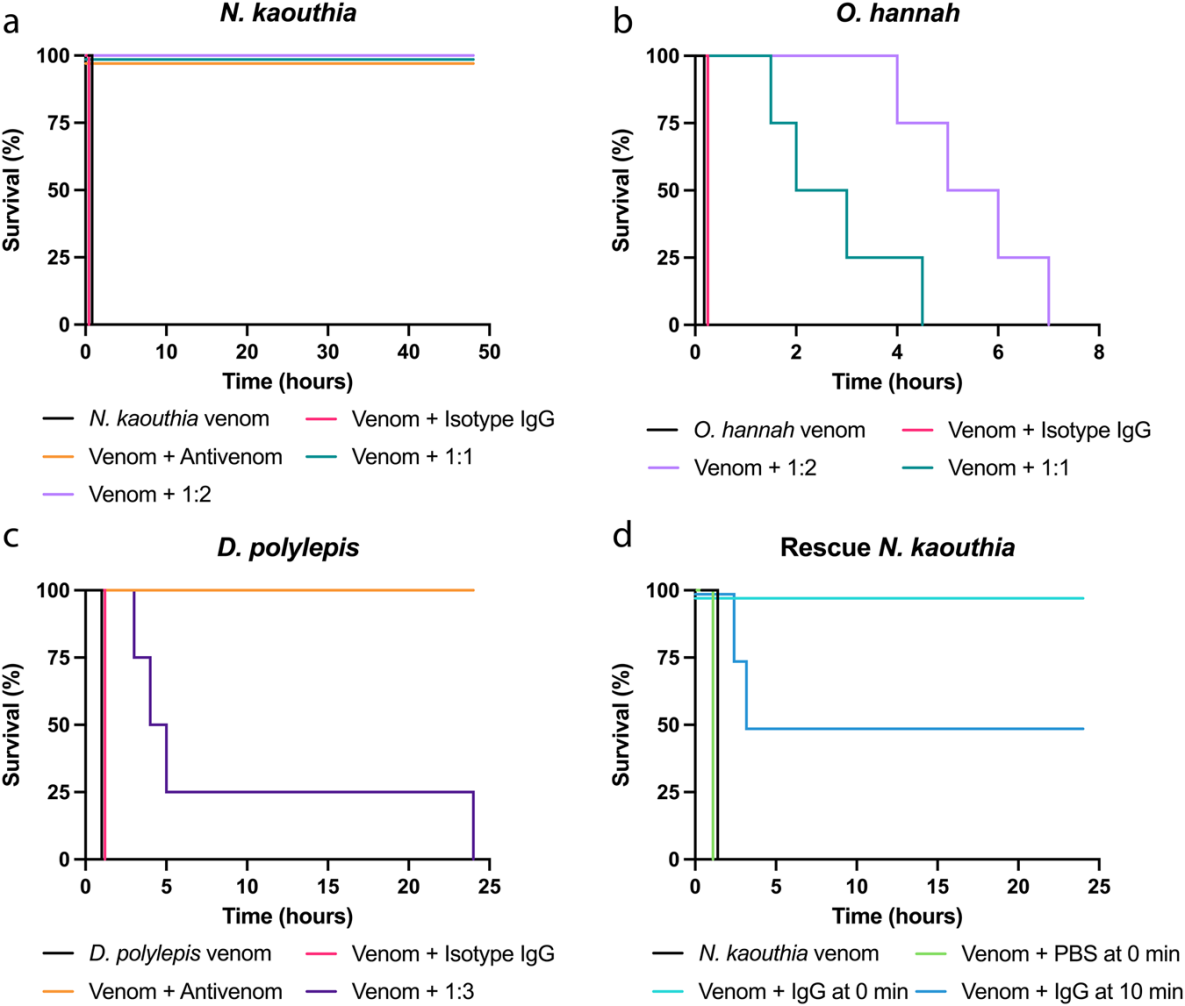
Kaplan–Meier survival curves for envenomed mice treated with the antibody 2554_01_D11. **a**–**c** Mixtures containing 2 LD_50_s of venom of either *N. kaouthia*, *O. hannah*, or *D. polylepis* were preincubated with the antibody, 2554_01_D11, at various neurotoxin:antibody ratios and then administered i.v. into groups of four mice. Controls included mice receiving venom alone, or venom incubated with either an irrelevant isotype antibody control or commercial horse-derived antivenoms (see Materials and Methods for details). Signs of toxicity were observed, and deaths were recorded for a maximum period of 48 h. **d** *N. kaouthia* venom at 2 LD_50_s was administered s.c. following by i.v. administration of IgG 2554_01_D11 either immediately or 10 min following venom injection. As controls, mice received either venom s.c. or venom s.c. followed by PBS i.v. immediately after envenoming. Signs of toxicity were observed, and deaths were recorded for 24 h. Mice receiving antibody immediately following venom administration had a dose of 1:2.5 toxin to antibody molar ratio, while mice receiving antibody 10 min post venom administration had an antibody dose of 1:2 toxin to antibody molar ratio.

Then, snake venoms from three different species belonging to three genera, one from Africa, i.e., *D. polylepis*, and two from Asia, *i.e., N. kaouthia* and *O. hannah*, were included. Notably, each of these venoms contains a substantial amount of long-chain α-neurotoxins (a relative abundance of 13.2%^25^, 55%^24^, and ∼20%^38^, respectively). Two LD_50_s of each venom were preincubated with 2554_01_D11 in a 1:1 and 1:2 toxin:antibody molar ratio for *N. kaouthia* (Fig. 6a) and O*. hannah* (Fig. 6b) or a 1:3 toxin:antibody molar ratio for *D. polylepis* (Fig. 6c) before being administered i.v. to the mice. As controls, mice were injected with venom alone, venom preincubated with commercial antivenoms of known efficacy against the venoms (except in the case of *O. hannah*, where no antivenom was available), or venom preincubated with an antibody isotype control.

The results of the studies demonstrated that all mice in the venom-only control group, as well as the mice receiving venom preincubated with the isotype control antibody, died within the first hour after the challenge, with evident signs of limb paralysis and respiratory difficulty. As expected, mice receiving *N. kaouthia* or *D. polylepis* venoms preincubated with commercially available antivenoms survived for the entire observation period, and no signs of neurotoxicity were observed. In experiments where mice were injected with venoms incubated with 2554_01_D11, results varied depending on the venom. In the case of *N. kaouthia* venom, complete neutralization was observed at both toxin:antibody molar ratios, since mice survived during the 48-hour observation time. Moreover, the mice showed no signs of neurotoxicity, i.e., limb paralysis or respiratory difficulty during the whole period. In the case of *O. hannah*, there was a dose-dependent delay in the time of death compared to controls receiving venom alone (Fig. 6b). Likewise, a delay in the time of death was observed in the case of *D. polylepis* venom (Fig. 6c).

Next, the ability of the antibody to abrogate the lethality of *N. kaouthia* venom in rescue-type experiments was assessed. For this, the subcutaneous (s.c.) route of venom injection was used to more closely reproduce the actual circumstances of envenoming. The LD_50_ estimated by the s.c. route was 10.3 µg (95% confidence interval: 5.0–16.8 µg). Mice were challenged by the s.c. route with a dose of venom corresponding to 2 LD_50_s, i.e., 20.6 µg, followed by the i.v. administration of the 2554_01_D11 antibody in a volume of 100 µL at a molar ratio of 1:2.5 or 1:2.0 (toxin:antibody). Control mice injected with venom-only died within 40–60 min, with evident signs of limb and respiratory paralysis. When the antibody was administered immediately after venom injection, all mice survived the 24 h observation period and showed no evidence of limb or respiratory paralysis. When the antibody was provided 10 min after venom injection, two out of four mice died, but there was a delay in the time of death (150 and 180 min). The other two mice survived the 24 h observation time and did not show signs of paralysis (Fig. 6d).

## Discussion

Here, we demonstrate that an antibody discovered from a naïve human library with limited cross-reactivity to other α-neurotoxins and without the ability to prevent lethality induced by α-cobratoxin in mice can be improved by light chain-shuffling, resulting in enhanced affinity, potency, and cross-neutralization capacity.

The most promising antibody we discovered, 2554_01_D11, bound seven long-chain α-neurotoxins deriving from five snakes of four genera distributed across both Asia and Africa. Notably, cross-reactive binding was detected despite sequence alignment of the seven α-neurotoxins revealing substantial differences in sequence identity, with an overall identity of only 31%. This is likely because, despite the low overall identity, a total of 29 positions in the toxin sequences contain identical amino acid residues across all seven α-neurotoxins, including most of the residues previously identified as playing a significant role in the binding between α-cobratoxin/α-bungarotoxin and the nAChR^39^. Specifically, these amino acid residues include Trp25, Cys26, Asp27, Ala28, Phe29, Cys30, Arg33, Lys35, and Arg36/Val39 (α-cobratoxin/α-bungarotoxin) on loop II and Phe65/Val39 (α-cobratoxin/α-bungarotoxin) on the C-terminus, where a single mutation of one of these residues has been shown to cause a more than five-fold decrease in affinity to the nAChR^40^. Furthermore, high-density peptide microarray analysis previously suggested that positions 22–27 and 36–46 represent linear B-cell epitopes for antibodies to long neurotoxin 2 from *N. melanoleuca*, recognized by one of the most effective antivenoms available (SAIMR, produced by SAVP), like positions 24–30, 32, and 39–50 do for α-elapitoxin^37^. This emphasizes the potential importance of Trp25, Cys26, Asp27, Ala28, Phe29, Cys30, and Arg36 for the ability of antibodies to recognize this toxin. Together, these findings present a plausible explanation for the broad cross-reactivity we observed for 2554_01_D11 and indicates the importance of epitope similarity (as opposed to overall sequence identity) in the pursuit of cross-reactive antibodies^4^. However, the boundaries of the cross-reactivity of 2554_01_D11 have not been established in this study, as all long-chain α-neurotoxins investigated were recognized by the antibody. Future work aiming to investigate the boundaries of cross-reactivity could include testing the antibody for binding to long-chain α-neurotoxins from other snakes, such as *B. candidus*^41^, *B. fasciatus*^41^, or *N. haje legionis*^42^. Such studies could potentially provide general cues to how antibody cross-reactivity can be optimized for antibodies targeting toxins and similar antigens.

In addition to cross-reactive binding, we also demonstrated the broad neutralizing potential of 2554_01_D11. In vivo studies showed that lethality induced by three snake venoms of different genera distributed across both Asia and Africa was either prevented or delayed by the antibody, 2554_01_D11. For *N. kaouthia* venom, the antibody was able to completely prevent lethality in envenomed mice with no signs of neurotoxicity even at the lowest tested toxin:antibody molar ratio of 1:1. Moreover, this antibody was able to neutralize lethality induced by the venom of *N. kaouthia* in rescue-type experiments, which more closely resemble the actual circumstances of envenoming^43^. Complete neutralization was achieved when the antibody was administered immediately after the venom challenge, and even after a delay of 10 min between venom and antibody administration, 2 out of 4 mice survived and death was delayed in the other two.

Despite the antibody 2554_01_D11 possessing very similar in vitro affinities to α-cobratoxin and α-elapitoxin from *D. polylepis*, the antibody was unable to prevent in vivo lethality induced by *D. polylepis* venom, even at the tested toxin:antibody molar ratio of 1:3. Survival was, however, prolonged by several hours, suggesting that the antibody provided partial neutralization of the venom. These results are perhaps not surprising, as the venom of *D. polylepis* is more complex than that of *N. kaouthia*, and it is well-established that toxins other than long-chain α-neurotoxins (i.e., short-chain α-neurotoxins and dendrotoxins) play important roles in the toxicity of *D. polylepis* venom^25^. Where lethality of *N. kaouthia* venom is mainly attributed to the high content of long-chain α-neurotoxins, the short-chain α-neurotoxins present in *D. polylepis* venom have been estimated to contribute approximately one-third of the toxicity of the venom^25^. Thus, even if all long-chain α-neurotoxins were neutralized in the venom, neutralization of short-chain α-neurotoxins and possibly even dendrotoxins could still be necessary to prevent venom-induced lethality. As the affinity of the antibody to α-elapitoxin was almost identical to that of α-cobratoxin, we speculate that the antibody, 2554_01_D11, might be able to neutralize the effects of the long-chain α-neurotoxins from *D. polylepis* but that the mice eventually die due to lethality induced by short-chain α-neurotoxins and possibly dendrotoxins. Similarly, the lethal effects of the venom of *O. hannah* were only delayed, probably since this venom also consists of a mixture of both long and short-chain α-neurotoxins.

In this study, monoclonal IgG antibodies with broad cross-reactivity to different long-chain α-neurotoxins were discovered, and an epitope binning study revealed that the cross-reactive antibody 2554_01_D11 bound the same or an overlapping epitope to the previously reported antibody 2552_02_B02, which only recognizes α-cobratoxin^15^. In the future, determination of the structure of the two antibodies in complex with α-cobratoxin might provide further insight into how two antibodies binding to the same or overlapping epitope with similar affinity can display such different levels of cross-reactivity.

When comparing the neutralizing capacities of the two antibodies, 2554_01_D11 and 2552_02_B02, another noteworthy observation emerges. Antibody 2554_01_D11 possessed significantly higher efficacy in neutralizing *N. kaouthia* venom in vivo than what was reported for 2552_02_B02^15^. Whereas 2554_01_D11 neutralized all signs of neurotoxicity at the lowest tested dose of 1:1 toxin to antibody molar ratio, 2552_02_B02 only prevented lethality induced by *N. kaouthia* venom in 3 out of 4 mice at a 1:4 molar ratio, with mice showing clear signs of neurotoxicity. These results were especially remarkable, as the two antibodies performed similarly both in electrophysiological in vitro neutralization assays and had similar affinities to α-cobratoxin (490 pM for 2552_02_B02 and 1.78 nM for 2554_01_D11). Except for the difference in cross-reactivity profiles, the only significant difference between the two antibodies was in their developability profiles. In these developability assessment assays, 2554_01_D11 performed similarly to Aliricumab (control for good developability), whereas 2552_02_B02 performed comparably to Bococizumab (control for poor developability)^44^. It is thus possible that this difference in self-association and interaction with the SEC column seen in these assays may correlate with different pharmacokinetic or pharmacodynamic properties of the two antibodies, which could explain their contrasting performance in vivo. This suggests that detailed developability characterization should be included as part of early discovery to maximize the in vivo efficacy and clinical success of recombinant antivenoms. Furthermore, whilst this study focuses on α-neurotoxins, we hypothesize that similar in vitro strategies for affinity maturation and increased cross-reactivity could find utility for other classes of toxins comprising clusters of similar isoforms (e.g., phospholipase A_2_s).

In conclusion, this study demonstrates the utility of combining cross-panning strategies in phage display with affinity maturation using chain-shuffling for the development of high-affinity human monoclonal IgG antibodies that show broadly-neutralizing effects against neurotoxic elapid snake venoms in vitro and in vivo. Such antibodies might be useful for designing future envenoming therapies, but more importantly, the pipeline presented here could also be exploited for the development of broadly-neutralizing antibodies against other targets of medical importance. These targets could include toxins from venomous animals other than snakes, but also hypervariable and mutating antigens from infectious bacteria, viruses, and parasites, or even neoepitopes in noninfectious diseases.

## Methods

Protocols for in vivo experiments were approved by the Institutional Committee for the Use and Care of Animals (CICUA), University of Costa Rica (approval number CICUA 82–08).

### Toxin preparation

α-cobratoxin (L8114), α-bungarotoxin (L8115), and whole venoms from *N. kaouthia* (L1323), *N. melanoleuca* (L1318), *D. polylepis* (L1309), and *O. hannah* (L1357) were obtained from Latoxan SAS, France. Venom fractions containing long α-neurotoxins (Dp7 from *D. polylepis* and Nm8 from *N. melanoleuca*) were isolated from crude venom by fractionation using RP-HPLC (Agilent 1200). Venoms were fractionated using a C18 column (250 × 4.6 mm, 5 μm particle; Teknokroma), and elution was carried out at 1 mL/min using Solution A (water supplemented with 0.1% TFA) and a gradient towards solution B (acetonitrile supplemented with 0.1% TFA): 0% B for 5 min, 0–15% B over 10 min, 15–45% B over 60 min, 45–70% B over 10 min, and 70% B over 9 min^25,31^. Fractions were collected manually and evaporated using a vacuum centrifuge. Toxins were dissolved in phosphate buffered saline (PBS: 137 mM NaCl, 3 mM KCl, 8 mM Na_2_HPO_4_.2H_2_O, 1.4 mM KH_2_PO_4,_ pH 7.4) and biotinylated by amine coupling using a 1:1 molar ratio of α-cobratoxin:EZ-Link™ NHS-PEG_4_-Biotin reagent (Thermo Scientific, A39259). For the remaining toxins, a 1:1.5 toxin:biotin molar ratio was used. Free biotin was removed using 4 K MWCO ultracentrifugation membranes (Amicon Ultra-4, UFC8000324) in accordance with the de-salting protocol in the manufacturers guidelines. Following purification, the degree of biotinylation was determined using MALDI-TOF in an Ultraflex II TOF/TOF spectrometer (Bruker Daltonics).

### Library generation using chain-shuffling

Light chain-shuffled libraries containing the heavy chain variable domain of the 368_01_C05, diversified with a naïve repertoire of light variable domains, were created by subcloning the 368_01_C05 heavy chain variable domain into pSANG4 phage display vectors containing naïve kappa and lambda light chain germlines. The heavy chain was prepared by PCR amplification from the pSANG10-3F expression vector using the Platinum SuperFi Green polymerase (Thermo Fisher Scientific, 12359010), and sub-cloned into the phagemid vectors using *XhoI* (NEB, R0146S) and *NcoI* (NEB, R3193L) restriction enzymes. Ligations were performed overnight at 16 °C using 500 ng of light chain vector and a 4-fold excess of heavy chain. Each library was purified using a MiniElute purification kit (Qiagen, 28006), eluted in 10 µL nuclease free water (Thermo Scientific, 10977035), and transformed into one aliquot electrocompetent TG1 cells (Lucigen, 605022) using a 0.2 cm Gene Pulser Cuvette (BioRad, 1652086). The library was immediately transferred into pre-warmed recovery medium (Corning, 354253) and incubated for 1 h at 37 °C, 220 rpm. Cells were pelleted, re-suspended in 500 µL of recovery media, plated on 2TYGA (2TY, 2% glucose, 50 µg/mL ampicillin), and incubated overnight at 30 °C. The library size was estimated based on counting the number of individual colonies from a dilution series of the library. The kappa library size was estimated to 1.67 × 10^8^ and the lambda library to 1.01 × 10^8^ individual transformats respectively. Colony PCR revealed that 96% of the transformants had successful insert of the heavy chain. The library was re-suspended in 2TYGA media containing 25% glycerol and left to homogenize for one hour with end over end rotation at room temperature before being stored at −80 °C.

### Library rescue, solution-based phage display selection, and polyclonal DELFIA

Phages were rescued from the light chain-shuffled libraries by first diluting the cells to an OD_600_ of 0.1 in 50 mL of pre-warmed 2TYGA and incubating at 37 °C, 280 rpm. This equated to enough cells to obtain a 10-fold coverage of the library size. Once the cells had reached OD_600_ = 0.5, a 10-fold excess of proteolytic sensitive helper phage^45^ was added for 1 h at 37 °C, 150 rpm to allow for infection. Cultures were centrifuged at 3,200 rpm for 2 min, the supernatant discarded, and cells were re-suspended in 2TYKA (2TY, 50 µg/mL kanamycin, 100 µg/mL ampicillin) media and the phages propagated overnight at 25 °C, 280 rpm. A TG1 colony was picked from a pre-prepared plate and used to inoculate 5 mL of 2TY media and incubated overnight at 30 °C, 280 rpm. The next day, the supernatant was obtained by centrifuging the overnight culture for 10 min, 10,500 × *g* at 4 °C, and a 1 mL volume of supernatant, sufficient for both the selection with antigen and a no-antigen reference, was spun under the same conditions to remove any residual cells and used for selection. At this point, 125 µL of each kappa and lambda library was combined into a 250 µL volume and blocked in a final concentration of 3% MPBS (PBS + 3% w/v milk: VWR, A0830.1000), in parallel with 80 µL streptavidin-coated Dynabeads (Fisher Scientific, M-280), for one hour with end over end rotation. Streptavidin-specific phages were de-selected by adding 80 µL of the blocked streptavidin beads to the blocked library for one hour with end over end rotation. The library with streptavidin beads was placed on a magnetic rack, and the supernatant containing non-streptavidin specific phages was transferred to a clean Eppendorf tube. Biotinylated long neurotoxin, diluted in 3% MPBS, was added to the de-selected library at a final concentration of 10 nM and incubated as described for previous steps followed by addition of streptavidin beads. Therafter, a KingFisher Flex (Thermo Scientific, 711-82573) system was used to wash the beads with biotinylated toxin and bound phages 3 times with PBST (PBS + 0.1% tween) and PBS before elution using 200 µL, 1 mg/mL trypsin (Sigma-Aldrich, T9201-500MG) prepared in 50 mM Tris, 1 mM CaCl_2_, pH 8.0 buffer. 200 µL of the eluted phages was used to infect 5 mL of TG1 cells grown to an OD_600_ of 0.5, for 1 h at 37 °C, 150 rpm. Cells were subsequently spun for 10 min at 2000 g and re-suspended in 200 µL of media before plating. In addition, dilution plates ranging from 5–500,000-fold dilution of the supernatant were plated to determine the background and enrichment of antigen specific phages. The next day, colonies on the output plate were scraped and resuspended in 2TYGA supplemented with 25 % glycerol and the cells were homogenized with end-over end rotation for several hours. OD_600_ was measured, and the cells were stored at −80 °C until subsequent rescue and further rounds of selection. Two consecutive rounds to enrich for cross-reactivity were performed by cross-panning between α-cobratoxin and α-elapitoxin. The lead 2554_01_D11 clone originated from a selection strategy using 1 nM and 100 pM of antigen in the second and third round respectively.

To assess the polyclonal output of the individual selections, the binding was measured to biotinylated α-cobratoxin and biotinylated α-elapitoxin (both 60 µL, 5 µg/mL) captured on coated streptavidin (60 µL, 10 µg/mL). MPBS and streptavidin were included as negative controls. Black MaxiSorp plates (Nunc) were used and coating was performed overnight at 4 °C. The generation of phages from each selection round was performed as previously described for phage display. The prepared phage supernatants were diluted 100-fold in 3% MPBS and blocked alongside the immobilized antigen for 1 h at room temperature. With three washes in PBST and PBS between each incubation step, 60 µL of phages were first incubated for 1 h to allow for binding. Thereafter, bound phages were detected by the addition of 60 µL, 1 µg/mL, of anti-M13 mouse antibody (GE Healthcare) prepared in 3% MPBS. Finally, anti-M13 was detected using 0.5 µg/mL of an anti-mouse antibody conjugated to europium (Perkin Elmer) diluted in 3% MPBS), incubated for 30 min, followed by 100 µL of DELFIA enhancement solution (Perkin Elmer). The signal readout was performed by time resolved fluorescence (TRF) with 320 nm excitation and 615 nm emission wavelengths.

### Subcloning, screening, and sequencing of scFvs

The genes encoding the scFvs from five of the obtained selection outputs (representing different cross-panning strategies) were PCR amplified using M13leadseq (AAATTATTATTCGCAATTCCTTTGGTTGTTCCT) and Notmycseq (GGCCCCATTCAGATCCTCTTCTGAGATGAG) primers and subcloned into the pSANG10-3F expression vector using the *Nco*I and *Not*I restriction enzymes. Following transformation into the *E. coli* strain BL21 (DE3) (New England Biolabs), 184 individual colonies were picked from each selection and used for expression of soluble scFv. Cells were first grown at 30 °C, 200 rpm, overnight in 150 µL of 2TYKA media containing 2% glucose. Expression was then induced in 96 well polypropylene microtiter plates (Greiner Bio-One) containing 150 µL of autoinduction media and incubated overnight at 30 °C, 800 rpm with 80% humidity. Binding of the scFv was assessed in both a direct and an expression-normalized DELFIA. For direct binding, MaxiSorp plates were coated overnight with streptavidin (60 µL, 10 µg/mL). The next day, following washing, biotinylated α-cobratoxin and α-elapitoxin (60 µL, 5 µg/mL) diluted in 3% MPBS were added and incubated for one hour. To assess scFv binding, 25 µL supernatant from the harvested overnight cultures was transferred to each well containing an equal volume of 6% MPBS for one hour to permit binding. Bound scFv was detected with an anti-FLAG M2 antibody (Sigma-Aldrich) conjugated with europium (PerkinElmer), produced in house according to the manufacturers guidelines, in the steps described above for the polyclonal DELFIA. In addition, an expression-normalized DELFIA was run. Here, black MaxiSorp plates were coated overnight with anti-FLAG (Sigma) (60 µL, 2.5 µg/mL). The next day, following washing, 25 µL supernatant mixed with 25 µL 6% MPBS was incubated for one hour at room temperature. After washing, 60 µL of 10 nM biotinylated α-cobratoxin and α-elapitoxin diluted in 3% MPBS was left to bind for one hour. Bound antigen was finally detected using 0.2 ng/µL of europium-conjugated streptavidin diluted in DELFIA assay buffer (Perkin Elmer), followed by enhancement solution and recording of emission as described above. Based on the signal intensity, the top 203 clones were cherry-picked and sequenced (Eurofins Genomics sequencing service) using the S10b primer (GGCTTTGTTAGCAGCCGGATCTCA). The antibody frameworks and the CDR regions of the light chains were annotated using Geneious Biologics (Biomatters), and 67 clones were identified as unique based on light chain CDR3 regions.

### Reformatting and screening of IgG and Fab formats

A total of 62 clones were selected for reformatting into human IgG1 and Fab format. The *V*_H_ and *V*_L_ domains were subcloned into a pINT12 vector for Fab expression and pINT3 vector for IgG expression. Each vector had the respective heavy chain constant domains and light chain constant domain pre-cloned for each format. The individual variable domains were PCR amplified from the pSANG10-3F expression vector using pSang10_pelB (CGCTGCCCAGCCGGCCATGG) and HLINK3_R (CTGAACCGCCTCCACCACTCGA) for the V_H_ and LLINK2_F (CTCTGGCGGTGGCGCTAGC) and 2097_R (GATGGTGATGATGATGTGCGGATGCG) for the V_L_. The PCR amplicons were prepared by digestion with *Nco*I and *Xho*I (V_H_ digestion) and *Nhe*I and *Not*I (V_L_ digestion) endonucleases. A four-part ligation including both variable domains, either the pINT3 or pINT12 vector containing the respective heavy chain constant domains, and a stuffer region containing the C_L_ and CMV promoter cut with *Nco*I and *Not*I, was performed with T4 DNA ligase (Roche, 10481220001). Each of the respective Fab and IgG formats were produced for screening at a 700 µL scale by transient mammalian expression using Expi293^TM^ cells (Thermo). After transfection using a ratio of 1 µg DNA/mL of Expi293^TM^ cells with ExpiFectamine^TM^ 293 (ThermoFisher, A14525) as per the manufacturers guidelines, transfected cells were incubated for 4 days in an orbital shaker at 37 °C, 5% CO_2_, 70% humidity with 1000 rpm shaking. Cells were harvested, and supernatants containing IgGs were purified with protein A using MabSelect SuRe (Neo Biotech, NB-45-00036-100), and Fabs using anti-C_H_1 resin (Thermo Scientific, 194320010). A volume of 10 $\times$ PBS sufficient to give a 1 $\times$ PBS final concentration was added to each well of supernatant. For IgG containing supernatants, 100 µL of protein A diluted 4-fold in PBS was added to each well and incubated overnight at 4 °C. Likewise, Fab supernatant was incubated overnight with 100 µL of anti-C_H_1 resin diluted 5-fold in PBS. Supernatants were transferred to a Unifilter membrane (GE Healthcare, 7700-2804) and loaded onto a 96 deep well microplate. In centrifugation intervals of 1 min, 1000 × *g*, the flow through was removed, then the resin was washed twice with 500 µL PBS, and antibody was eluted with 75 µL, 0.2 M Glycine, pH 2.6 into a new plate containing 25 µL of 2 M Tris pH 8.0 neutralization buffer. Antibodies were desalted using Zeba Spin Desalting plates (Thermo Scientific, 89808) into PBS.

Produced clones were selected for further developability characterization by assessing their binding strength as full length IgGs and Fabs to a one-spot concentration of α-cobratoxin, α-elapitoxin, and Nm8, by a capture DELFIA assay. Black Maxisorp plates (Nunc) were coated with 50 µL, 2.5 µg/mL anti-C_H_1 antibody (Hybridoma Reagent Laboratory, HP6046P) or anti-hIgG (Jackson ImmunoResearch, 109-005-098) and incubated overnight at 4 °C. The next day, after washing the plates three times in PBS and blocking for an hour in 3% MPBS, 50 µL of supernatants diluted 4-fold in 4% MPBS was added to each well and incubated for 1 h at room temperature. After three washes in PBS and PBST, antigen (50 µL, 1 nM) prepared in 3% MPBS was added to test wells. As a control, a 1:500 dilution of streptavidin conjugated with europium in DELFIA assay buffer was added. After 1 hour, the test wells were washed, and streptavidin conjugated europium was added as for the control wells for 30 min. After a final wash, 50 µL of DELFIA enhancement solution was added, plates were placed on a shaker for 5 min, and binding was detected by time resolved fluorescence measured on a PHERAstar FSX (BMG, Labtech) system using excitation and emission wavelengths of 320 and 615 nm.

Six antibodies (2551_01_A12, 2554_01_D11, 2558_02_G09, 2551_01_B11, 2555_01_A04, and 2555_01_A01) and nivolumab were selected for further in vitro characterization and produced as IgGs at either a 300 mL or 500 mL scale. The production conditions and sample preparation for purification were the same as for the small-scale production, with the exception that the supernatants were first filtered using a 0.45 µm filter and purified using an Äkta Pure system (Cytiva) with a HiTrap^TM^ 5 mL MabSelect^TM^ Prism A column (Cytiva, GE17549854). Antibodies were eluted with 1.6 mL, 0.1 M citrate buffer, pH 3.0 into 300 µL, 2 M Tris pH 8.0 neutralizing solution. The eluate was buffer exchanged into 222 mM sucrose, 6.44 mM L-Histidine, 4.77 mM L-Histidine HCl, 0.0003% polysorbate 80 using P50 gel filtration columns (CentriPure, CP-0113-Z010.0-001). Antibodies were concentrated to 4–8 mg/mL using pre-rinsed Amicon® Ultra-4 Centrifugal Filter Units with a 50 KDa cutoff (Millipore, UFC8050) and flash frozen in liquid nitrogen for long term storage at −80 °C.

### Developability characterization

To aid in the selection of the top antibody candidates for further characterization, the biophysical behavior of the 62 reformatted clones in the IgG format was characterized using HPLC-SEC and AC-SINS. For HPLC-SEC, the purified antibodies were loaded onto a Superdex 200 Increase 5/150 column at a flow rate of 0.25 mL/min using an Agilent 1100 HPLC instrument. AC-SINS was performed to measure the self-propensity of antibodies. Gold nanoparticles were coated with a 0.4 mg/mL, 20 mM NaAc, pH 4.3 solution containing a 4:1 ratio of anti-human IgG Fc capture:non-capture antibodies. The antibody coating solution was mixed with the gold nanoparticles in a 1:9 volume ratio at room temperature for 1 h and the remaining sites blocked with a 0.1 µM final concentration of thiolated PEG. After filtering through a 0.22 µm PVDF membrane (Millex-GV, 13 nm, Millipore), the gold nanoparticles were present in the retentate, and were eluted in 1/10th the volume of PBS. The prepared gold nanoparticles (10 µL) were mixed with test antibody (100 μL, >50 ug/ml in PBS) at room temperature for 2 h in a polypropylene plate, after which they were transferred to a polystyrene UV transparent plate, centrifuged briefly, and data collected by measuring the absorbance from 510 to 570 nm in increments of 2 nm. The self-association propensity was calculated by importing and processing the raw absorbance as described perviously^32^.

### Surface plasmon resonance

The binding affinity of the corresponding Fab versions of the top six affinity matured antibodies as well as the parental clone to α-cobratoxin and α-elapitoxin was determined using surface plasmon resonance (SPR; BIAcore T100, GE Healthcare). α-elapitoxin and α-cobratoxin were immobilized to a target level of 20 response units (RU) by amine coupling to carboxymethylated dextran on a CM5 biosensor chip (Cytiva, BR100530). The biosensor surface was activated using 1-Ethyl-3-(3-dimethylaminopropyl) carbodiimide (EDC)/N-hydroxysuccinimide followed by an injection of 5 µg/mL of α-neurotoxin prepared in 10 mM NaOAc pH 4. A no antigen flow cell was allocated as a reference. After immobilization the surface was washed and de-activated using ethanolamine. Antibody Fab fragments were prepared in running buffer: 10 mM HEPES, 150 mM NaCl, and 3 mM EDTA, 0.05% P20 (HEPES), adjusted to pH 7.4, in a 3-fold dilution series from 81 to 390 pM. A flow rate of 40 µL/min was used throughout the experiment with an association time of 120 s and a dissociation time of 450 s for each Fab. The biosensor surface was regenerated using two consecutive 30 s injections of 10 mM Glycine, 4 M sodium chloride pH 2.0. Measurements were conducted using 5–7 analyte concentrations for each antibody and included a no antibody blank. The blank and reference flow cell backgrounds were subtracted in the BIAcore T100 Evaluation Software, a 1:1 Langmuir binding model and a global model was used for fitting of the data and calculations of kinetic parameters.

Epitope binning experiments were performed using a sandwich setup, whereby one antibody was immobilized on a CM5 sensor chip using amine coupling, prior to flowing α-cobratoxin with a competing antibody. The 2554_01_D11 Fab (10 µg/mL, 10 mM sodium acetate pH 5.0) was immobilized to a level of 450 RU, and 20 nM α-cobratoxin prepared in HEPES buffer (10 mM, HEPES, 150 mM NaCl, 50 mM MES, 0.05% P20, pH 7.4) was incubated with 200 nM of either test 2552_02_B02 Fab or control Fab. Dual binding was then measured by injecting the α-cobratoxin and Fab solution over the immobilized 2554_01_D11 flow cell for 120 s. The immobilized 2554_01_D11 Fab flow cell was used to measure the affinity to α-bungaratoxin, prepared in a 3-fold titration series from (2.1 µM to 9 nM) in HEPES buffer and regenerated as decribed above. The background subtraction and data processing was performed as described for α-elapitoxin and α-cobratoxin.

### Determining cross-reactivity using native mass spectrometry

#### Sample preparation

Venoms and antibody samples were fractionated and exchanged into 200 mM ammonium acetate by size exclusion chromatography (SEC). Briefly, 2–5 mg of whole venom was dissolved in 200 mM ammonium acetate^46,47^. Size exclusion was then performed on this whole venom solution using a Superdex Increase 200 10/300 GL column (Cytiva, Massachusetts, United States) pre-equilibrated with 200 mM ammonium acetate. Samples were collected and stored at 4 °C until use. Prior to analysis, aliquots of the venom and IgG 2554_01_D11 SEC fractions were mixed in a 1:1 ratio (*v*/*v*). The final concentration of the antibody was approximately 3 μM after mixing. The concentration of toxins in the SEC fractions was not adjusted prior to mixing with the antibody.

#### Native mass spectrometry

All mass spectrometry (MS) experiments were performed on a SELECT SERIES cyclic IMS mass spectrometer (Waters, Manchester, U.K.) which was fitted with a 32,000 *m/z* quadrupole, as well as an electron capture dissociation (ECD) cell (MSvision, Almere, Netherlands), the latter of which was situated in the transfer region of this mass spectrometer. Approximately 4 μL of sample was nanosprayed from borosilicate capillaries (prepared in-house) fitted with a platinum wire. Spectra were acquired in positive mode, with the *m/z* range set to 50–8,000. Acquisitions were performed for five minutes at a rate of 1 scan per second. The operating parameters for the MS experiments were as follows, unless otherwise stated: capillary voltage, 1.2–1.5 kV; sampling cone, 20 V; source offset, 30 V; source temperature, 28 °C; trap collision energy, 5 V; transfer collision energy, 5 V; and ion guide RF, 700 V. This instrument was calibrated with a 50:50 acetonitrile:water solution containing 20 μM cesium iodide (99.999%, analytical standard for HR-MS, Fluka, Buchs, Switzerland) each day prior to measurements.

#### Top-down proteomics of toxins bound by 2554_01_D11

The toxin:antibody complexes were purified using SEC using the methods described above. Toxins were ejected from the protein complex during the MS experiments by setting the cone voltage to 160 V. The 5^+^ ions (most abundant charge state) of the ejected toxins were selected by tandem MS (MS/MS) and subjected to fragmentation by applying a trap voltage between 80 and 100 V as well as a transfer voltage between 20 and 50 V. Peptide sequence assignment was performed for 1^+^ fragmentation ions using the BioLynx package, which is a part of the MassLynx v4.1 software.

### Sequence alignment

Sequence alignment was performed in Clustal Omega^48^ and visualized in Jalview^49^ using α-cobratoxin (P01391) from *N. kaouthia*, α-elapitoxin (P01396) from *D. polylepis*, α-bungarotoxin (P60615) from *B. multicinctus*, long neurotoxin 2 (A8N285) from *O. hannah*, and long neurotoxin (P0DQQ2) and long neurotoxin 2 (P01388) from *N. melanoleuca*. Structures for each toxin were retrieved prioritizing high-resolution X-ray resolved structures and included the following: P01391 = 1CTX (2.8 Å, X-ray), P01388 = AF-P01388-F1 (AlphaFold2 predicted), P01396 = AF-P01396-F1 (AlphaFold2 predicted), P60615 = 1HC9 (1.8 Å, X-ray), A8N285 = AF-A8N285-F1 (AlphaFold2 predicted), and P0DQQ2 = AF-P0DQQ2-F1 (AlphaFold2 predicted). Structural alignment and root-mean-square deviation (RMSD) analysis were performed in ChimeraX^50^. Epitopes of P01388 and P01396 were identified using the STAB Profiles tool^37^ (https://venom.shinyapps.io/stab_profiles/).

### In vitro neutralization using electrophysiology (QPatch)

To determine the ability and potency with which the affinity matured clones 2551_01_A12 and 2554_01_D11, as well as the parental antibody 368_01_C05, were able to neutralize the effects of α-cobratoxin, whole-cell patch-clamp experiments were conducted using rhabdomyosarcoma cells (CCL-136, ATCC):

Planar whole-cell patch-clamp experiments were carried out on a QPatch II automated electrophysiology platform (Sophion Bioscience), where 48-channel patch chips with 10 parallel patch holes per channel (patch hole diameter ∼1 μm, resistance 2.00 ± 0.02 MΩ) were used.

The human derived Rhabdomyosarcoma RD cell line endogenously expresses the muscle-type nicotinic acetylcholine receptors (nAChR), composed of the α1, β1, δ, γ and ε subunits. The cells were cultured according to the manufacturer’s guideline and on the day of the experiment, enzymatically detached from the culture flask and brought into suspension.

For patching, the extracellular solution contained (in mM): 145 NaCl, 10 HEPES, 4 KCl, 1 MgCl_2_, 2 CaCl_2_, and 10 glucose, pH adjusted to 7.4 and osmolality adjusted to 296 mOsm and the intracellular solution contained (in mM): 140 CsF, 10 HEPES, 10 NaCl, 10 EGTA, pH adjusted to 7.3 and osmolality adjusted to 290 mOsm.

In the experiments, a nAChR mediated current was elicited by 70 µM acetylcholine (ACh, Sigma-Aldrich), approximately the EC_80_ value, and after compound wash-out, 2 U acetylcholinesterase (Sigma-Aldrich) was added to ensure complete ACh removal. The ACh response was allowed to stabilize over 3 ACh additions, before the 4th addition was used to evaluate the effect of α-cobratoxin (4 nM α-cobratoxin, reducing the ACh response by 80%), in combination with varying concentrations of IgG. α-cobratoxin and IgGs were co-incubated at least 30 min before application, and the patched cells were preincubated with α-cobratoxin and IgG for 5 min prior to the 4th ACh addition.

The inhibitory effect of α-cobratoxin was normalized to the full ACh response (4th response normalized to 3rd response), plotted in a non-cumulative concentration-response plot and a Hill fit was used to obtain EC50 values for each IgG. The data analysis was performed in Sophion Analyzer (Sophion Bioscience) and GraphPad Prism (GraphPad Software).

### In vitro cross-neutralization using electrophysiology (Qube 384)

To determine the broader cross-neutralizing potential of the top two affinity matured antibodies and the parent antibody, automated patch-clamp experiments using the Qube 384 electrophysiology platform (Sophion Bioscience) were conducted:

Planar whole-cell patch-clamp experiments were carried out on a Qube 384 automated electrophysiology platform (Sophion Bioscience), where 384-channel patch chips with 10 parallel patch holes per channel (patch hole diameter ∼1 μm, resistance 2.00 ± 0.02 MΩ) were used.

Both the RD cell line, the extracellular solution and the intracellular solution were similar to what was used in the QPatch experiments. Again, a nAChR mediated current was elicit by 70 µM ACh, and after compound wash-out, 2 U acetylcholinesterase was added to ensure complete ACh removal.

In the Qube 384 experiments, a 2nd ACh addition was used to evaluate the toxin effect (in nM: α-cobratoxin 1.47 nM, α-elapitoxin 0.81, nM 8 14, nM3 10.30) in combination of varying concentrations of IgG. α-cobratoxin and IgGs were co-incubated at least 30 min before application, and the patched cells were preincubated with α-cobratoxin and IgG for 5 min prior to the 2nd ACh addition.

The inhibitory effect of the toxins was normalized to the full ACh response and averaged in the group. The data analysis was performed in Sophion Analyzer (Sophion Bioscience) and Excel (Microsoft).

### Production of IgG for in vivo experiments

The variable chains (V_L_ and V_H_) were PCR-amplified from the pSANG10-3F vector and cloned into a single expression vector using the NEBuilder® cloning technique. The expression vector contained the constant domain sequences of the respective human IgG heavy chain (with LALA^51^ and YTE^52^ mutation) and human lambda light chain. After cloning and sequence verification the plasmid was purified using NucleoBond Xtra Midi EF (Macherey-Nagel) according to the manufacturer’s instructions.

A CHO-S cell line with pre-established landing pad (isoCHO-EP^53^) was cultivated in CD CHO medium, supplemented with 8 mM L-Glutamine and 2 μL/mL anticlumping agent at 37 °C, 5% CO_2_ at 120 rpm (shaking diameter 25 mm). The cell line was transfected with IgG expression vector and Cre-recombinase vector in 3:1 ratio (w:w) at a concentration of 10^6^ cells/mL using FreeStyle MAX transfection reagent (Thermo Fischer Scientific) according to the manufacturer’s recommendation. Stable cell pools were generated by adding 5 µg/mL blasticidin five days post-transfection, and after recovery (>95% viability), cells were single cell sorted using a fluorescence activated cell sorter. After expansion, the IgG producing cell line was seeded at 10^6^ cells/mL in 2 L complete culture medium and cultured for 144 h. Thereafter, cell cultures were harvested by centrifugation. The clear supernatant was loaded on a 25 mL MabSelect PrismA column (Cytiva) equilibrated with 20 mM sodium phosphate and 150 mM NaCl (pH 7.2). Elution was performed with 0.1 M sodium citrate (pH 3). Elution fractions were neutralized with 1 M Tris (pH 9), using 2 mL per 10 mL of elution fraction followed a buffer exchange to Dulbecco’s PBS using a HiPrep 26/10 desalting column. IgG was concentrated using an Amicon® Ultra-15 centrifugal filter unit (30 kDa NMWL), sterile filtered.

### Animals

Animal experiments were conducted in CD-1 mice of both sexes weighing 18–20 g (corresponding to 4–5 weeks old). Mice were supplied by Instituto Clodomiro Picado and experiments were conducted following protocols approved by the Institutional Committee for the Use and Care of Animals (CICUA), University of Costa Rica (approval number CICUA 82-08). Mice were provided food and water *ad libitum* and housed in Tecniplast Eurostandard Type II 1264C cages (L25.0 cm × W40.0 cm × H14.0 cm) in groups of 4 mice per cage. Animals were maintained at 18–24 °C, 60–65% relative humidity and 12:12 light-dark cycle.

### In vivo preincubation neutralization experiments

The in vivo neutralizing potential of 2554_01_D11 against α-cobratoxin and whole venoms of *N. kaouthia*, *D. polylepis*, and *O. hannah* was assessed by i.v. injection of IgG preincubated with toxin or venoms using groups of four mice per treatment. Mixtures of a constant amount of toxin or venom and various amounts of antibody were prepared and incubated for 30 min at 37 °C. Then, aliquots of the mixtures containing 2 LD_50_s of toxin or venoms (for α-cobratoxin 4.0 µg; for *N. kaouthia*, 9.12 µg; for *D. polylepis*, 25.8 µg; and for *O. hannah*, 40 µg) were injected into the caudal vein of mice using an injection volume of 150–200 µL. Control mice were injected with either toxin alone, venom alone, venom preincubated with an isotype control IgG or, in the cases of *N. kaouthia* and *D. polylepis* venoms, they were also preincubated with commercial horse-derived antivenoms known to be effective against these venoms. For *N. kaouthia* Snake Venom Antiserum from VINS Bioproducts Limited (batch number: 01AS13100), at a proportion of 0.2 mg venom per mL antivenom was used. For *D. polylepis*, Premium Serum and Vaccines antivenom (batch number: 062003), at a proportion of 0.12 mg venom per mL antivenom was used. These proportions were selected based on information on neutralization provided in the prospects of these products. In the case of *O. hannah* venom, no effective commercial antivenom was available and, therefore, this control group was not included.

IgG was injected using 1:1 and 1:2 α-neurotoxin:IgG molar ratios for α-cobratoxin, *N. kaouthia* venom and *O. hannah* venom and a 1:3 α-neurotoxin:IgG molar ratio for *D. polylepis* venom. In the case of venoms, for calculating molar ratios, based on toxicovenomic studies, it was estimated that 55% of *N. kaouthia* venom^24^, 13.2% of *D. polylepis* venom^25^, and 40% of *O. hannah* venom consisted of α-neurotoxins^54^. Following injection, animals were observed for signs of neurotoxicity, and survival was monitored for 48 h. The results are presented in Kaplan–Meier plots, generated with Prism v.9.4.1.

### In vivo rescue neutralization experiments

To assess whether the antibody was capable of neutralizing the venom of *N. kaouthia* in an experimental setting that more closely resembles the actual circumstances of envenoming, a rescue-type experiment was designed. For this, the s.c. route was used for injection of venom, while the antibody was administered i.v. First, the s.c., LD_50_ of *N. kaouthia* venom was estimated by injecting various doses of venom, diluted in 100 µL of PBS, into groups of four mice. Animals were observed for 24 h, deaths were recorded, and the LD_50_ was estimated by probits^55^. For neutralization experiments, groups of four mice first received a challenge dose of 20.6 µg of venom by the s.c. route, dissolved in 100 µL of PBS, corresponding to 2 LD_50_s. Then, antibody 2554_01_D11 was administered i.v. in the caudal vein, in a volume of 100 µL, either immediately or 10 min following venom administration. The amount of antibody administered was 535 µg (immediately) and 412 µg (10 min), corresponding to neurotoxin: antibody molar ratios of 1:2.5 and 1:2.0, respectively. Mice were observed for 24 h for the onset of neurotoxic manifestations, and times of death were recorded and presented in Kaplan-Meier plots, generated with Prism v.9.4.1.

### Statistics and reproducibility

No statistical method was used to predetermine the sample size. The experiments were not randomized. The investigators were not blinded to allocation during experiments and outcome assessment. All in vitro *data* was performed at least 4 times independently. Data were excluded for analysis based on the following quality filters (per site, each site records from 10 cells): Current minimum 500 pA and seal resistance minimum 50 XX.

### Reporting summary

Further information on research design is available in the Nature Portfolio Reporting Summary linked to this article.

## Supplementary information

Supplementary Information

Reporting Summary

## Data Availability

The data that supports the findings of this study are available from the corresponding authors upon reasonable request.
