# Supplementary material for: Discovery and optimization of a broadly-neutralizing human monoclonal antibody against long-chain α-neurotoxins from snakes
Source: Nat Commun. 2023 Feb 8;14:682. doi: 10.1038/s41467-023-36393-4 (PMC9908967; doi:10.1038/s41467-023-36393-4)
Supplement: Supplementary file 1 — Supplementary Information [file 41467_2023_36393_MOESM1_ESM.pdf]

Discovery and optimization of a broadly-neutralizing human monoclonal antibody against long-chain α-neurotoxins from snakes

Supplementary material

Table S1. Expression and developability data for 62 IgGs and parental IgG. The expression yield is provided for cultures in 96-well format. The AC-SINS shift reflects the IgG self-association propensity, and SEC analysis provides information on retention volume as well as the percentage of IgG monomers and dimers detected.

	Production	AC-SINS	SEC analysis		
**Antibody ID**	Yield (mg/L)	Shift (nm)	Retention volume (mL)	Monomer (%)	Dimer (%)
2551_01_A12	7.7	3	1.48	100.0	0.0
2554_01_D11	14.0	1	1.48	94.8	5.2
2552_02_B07	10.7	1	1.47	97.1	2.9
2558_02_G09	21.7	1	1.48	95.6	4.4
2551_01_A11	10.6	2	1.50	95.2	4.8
2554_01_E01	15.5	2	1.49	96.6	3.4
2551_01_B11	11.1	1	1.50	96.2	3.8
2554_02_F10	23.6	1	1.48	96.5	3.5
2551_01_A02	11.3	1	1.46	97.0	3.0
2552_01_G02	11.7	2	1.49	96.6	3.4
2554_01_E10	12.2	1	1.47	95.2	4.8
2554_02_F11	13.9	1	1.48	94.4	5.6
2555_01_A04	8.7	1	1.47	100.0	0.0
2555_01_A01	16.0	1	1.47	96.7	3.3
2554_02_G09	14.8	4	1.48	97.0	3.0
2558_01_E06	15.2	1	1.47	96.3	3.7
2554_01_E03	14.5	1	1.48	96.6	3.4
2554_01_C11	14.4	1	1.47	97.2	2.8
2554_01_D10	13.2	1	1.47	96.8	3.2
2554_01_E05	9.8	2	1.46	96.9	3.1
2555_02_D09	14.8	2	1.46	97.0	3.0
2555_02_D02	7.3	1	1.49	100.0	0.0
2555_01_B09	16.5	1	1.47	96.0	4.0
2554_01_D05	13.9	2	1.47	97.1	2.9
2554_01_C07	14.0	2	1.47	96.6	3.4

2558_01_E08	13.1	1	1.47	96.7	3.3
2554_02_G10	13.6	1	1.47	96.9	3.1
2558_02_F12	12.9	1	1.47	96.6	3.4
2555_02_D05	11.4	2	1.47	100.0	0.0
2551_02_E01	11.3	2	1.52	95.2	4.8
2558_02_H05	10.0	2	1.49	96.6	3.4
2558_02_G01	8.0	3	1.48	100.0	0.0
2558_02_G10	17.2	2	1.48	96.4	3.6
2551_01_B01	12.2	1	1.43	96.5	3.5
2554_02_H01	9.1	1	1.48	96.5	3.5
2554_02_G12	13.9	2	1.56	100.0	0.0
2554_01_D01	9.9	2	1.51	97.1	2.9
2551_01_A01	2.7	7	1.49	100.0	0.0
2554_02_G07	15.9	1	1.48	96.6	3.4
2554_02_F12	11.4	1	1.47	95.3	4.7
2555_01_B01	19.7	1	1.48	95.4	4.6
2558_01_E04	12.1	1	1.51	95.6	4.4
2558_02_H06	9.3	1	1.47	100.0	0.0
2558_01_E03	20.1	1	1.47	96.1	3.9
2558_01_D11	15.4	1	1.47	97.2	2.8
2558_02_G07	17.9	0	1.46	96.4	3.6
2555_01_B04	17.3	1	1.48	97.4	2.6
2558_02_G02	20.1	-1	1.48	96.2	3.8
2558_01_E02	10.7	2	1.48	100.0	0.0
2555_02_D06	21.4	0	1.46	96.9	3.1
2554_01_D09	7.3	2	1.46	100.0	0.0
2555_02_D07	17.2	0	1.48	96.2	3.8
2558_01_D12	15.6	1	1.47	95.9	4.1

2558_02_G03	14.4	1	1.49	91.1	8.9
2558_01_E10	16.7	0	1.48	96.6	3.4
2558_02_G04	17.6	0	1.46	97.3	2.7
2554_01_D04	3.8	3	1.47	100.0	0.0
2554_02_F04	18.9	-	1.49	95.5	4.5
2555_02_C08	16.2	-	1.55	97.2	2.8
2558_02_G05	15.1	-	1.47	97.0	3.0
2555_01_A08	12.3	-	1.48	96.1	3.9
2558_02_F10	6.9	-	1.47	100.0	0.0
368_01_C05	14.7	0	1.45	97.5	2.5

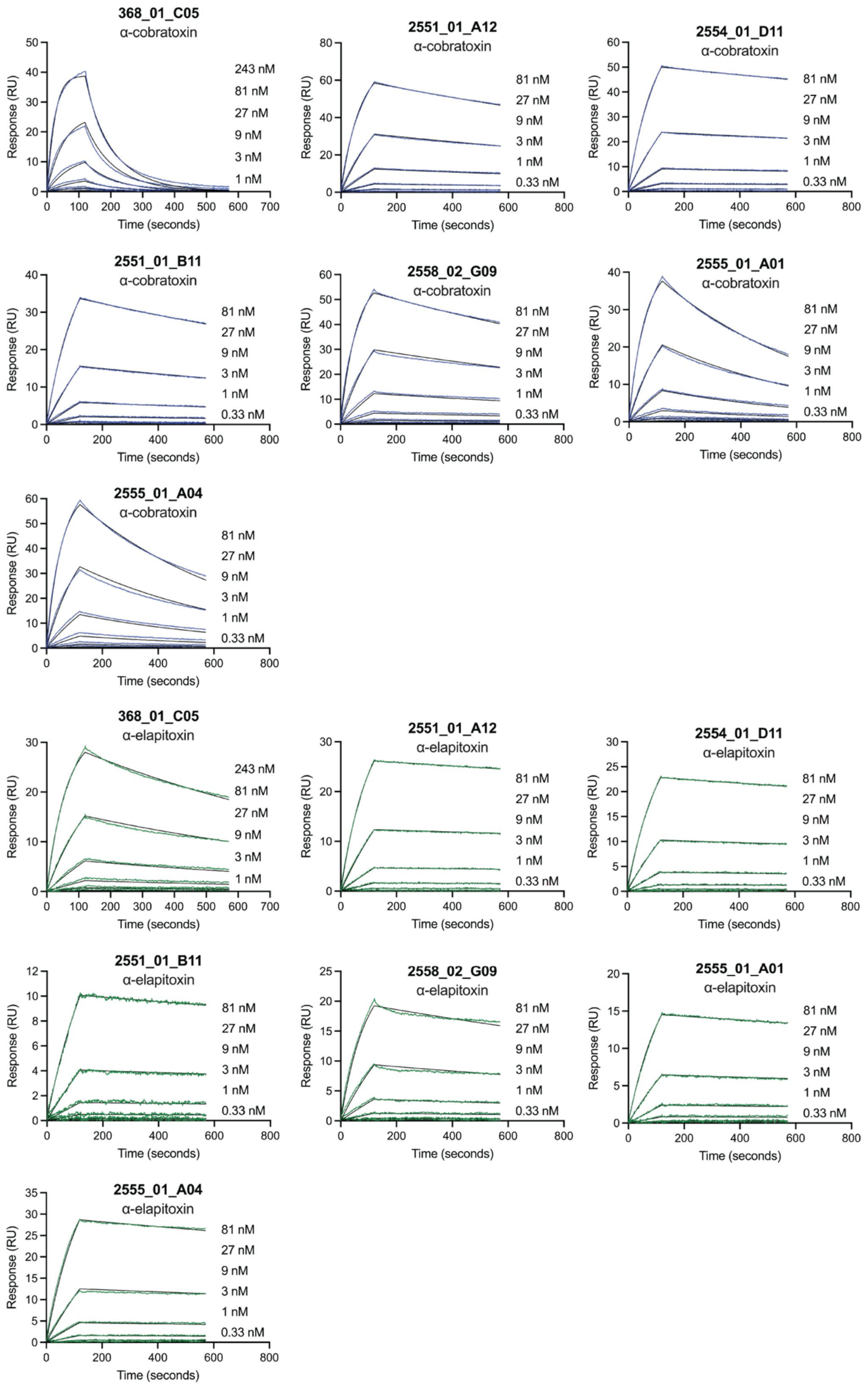
368_01_C05
α-cobratoxin
243 nM
81 nM
27 nM
9 nM
3 nM
1 nM
Response (RU)
Time (seconds)
2551_01_A12
α-cobratoxin
81 nM
27 nM
9 nM
3 nM
1 nM
0.33 nM
2554_01_D11
α-cobratoxin
2551_01_B11
α-cobratoxin
2558_02_G09
α-cobratoxin
2555_01_A01
α-cobratoxin
2555_01_A04
α-cobratoxin
368_01_C05
α-elapitoxin
2551_01_A12
α-elapitoxin
2554_01_D11
α-elapitoxin
2551_01_B11
α-elapitoxin
2558_02_G09
α-elapitoxin
2555_01_A01
α-elapitoxin
2555_01_A04
α-elapitoxin

9

Fig. S1. Affinity measurements using surface plasmon resonance. Sensorgrams illustrating affinity measurements of the top six affinity matured antibodies as well as the parent on α-cobratoxin and α-elapitoxin immobilized on a CM5 sensor.

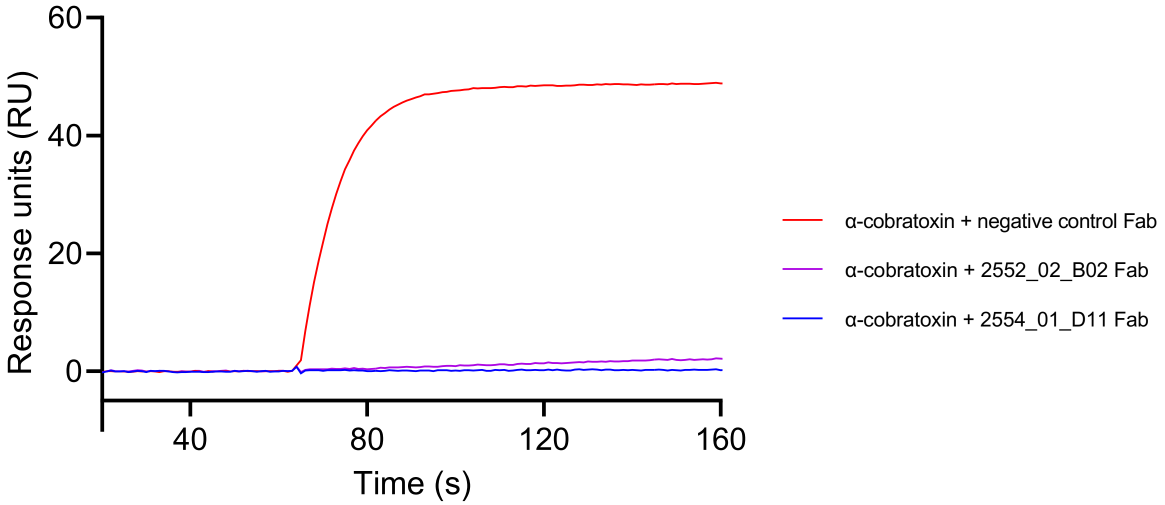

Fig. S2. Epitope binning of 2554_01_D11 and 2552_02_B02 anti-α-cobratoxin Fabs by surface plasmon resonance. Sensorgrams of α-cobratoxin interacting with immobilized 2554_01_D11 Fab in the presence of 2552_02_B02 Fab. Here, 200 nM of Fab was preincubated for 30 min with 20 nM α-cobratoxin before being flowed over immobilized 2554_01_D11 for 120 s. A non-α-cobratoxin-specific Fab and the 2554_01_D11 Fab were included as negative and positive controls, respectively.

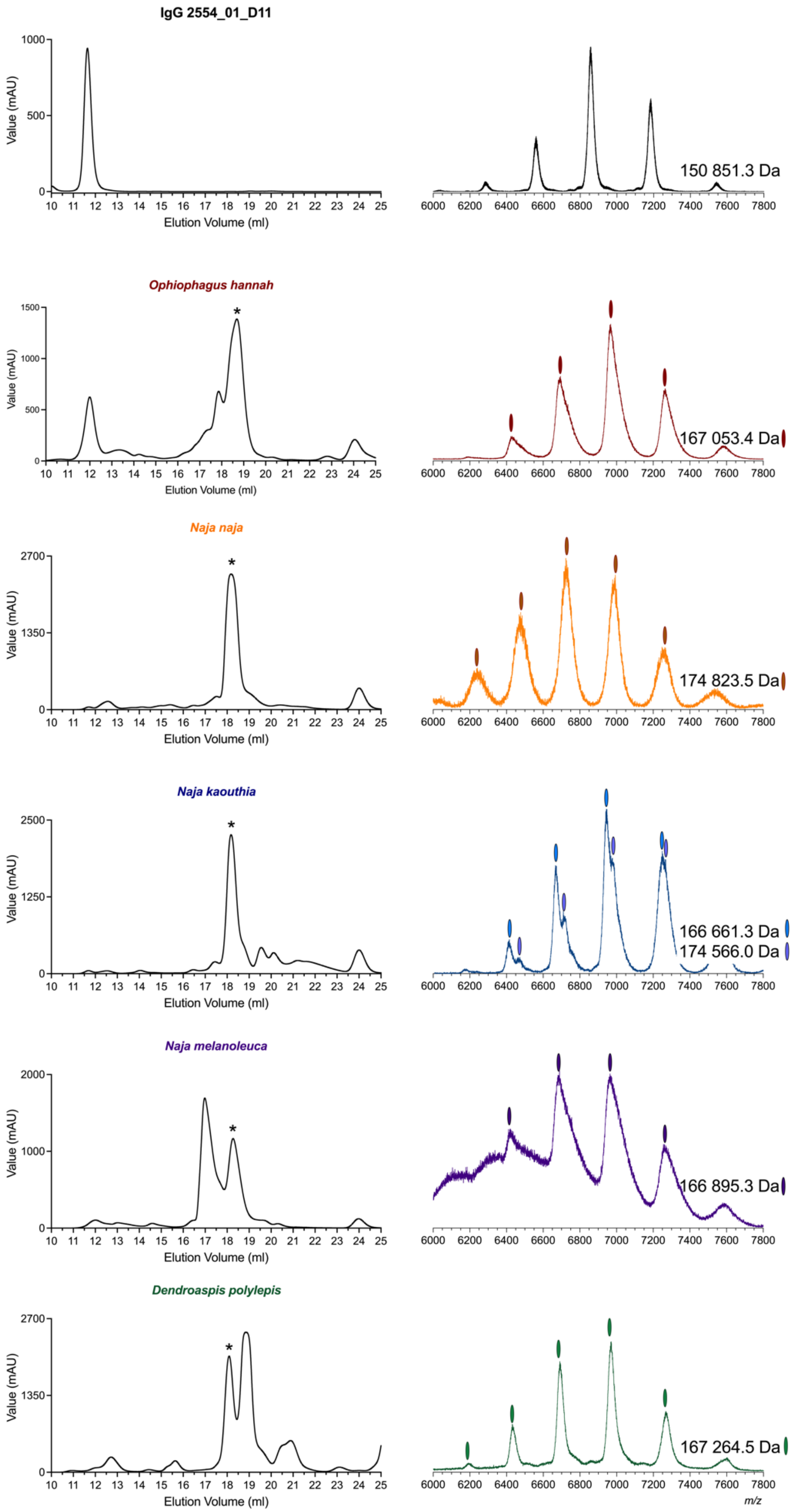
IgG 2554_01_D11
Ophiophagus hannah
Naja naja
Naja kaouthia
Naja melanoleuca
Dendroaspis polylepis
Value (mAU)
Elution Volume (ml)
m/z
150 851.3 Da
167 053.4 Da
174 823.5 Da
166 661.3 Da
174 566.0 Da
166 895.3 Da
167 264.5 Da

23

Fig S3. Size exclusion chromatograms of the snake whole venoms and native mass spectra of toxin:antibody complexes. Size exclusion chromatograms of IgG 2554_01_D11 and five featured venoms accompanied by native mass spectra of IgG 2554_01_D11 mixed with the toxin fractions marked with an asterisk from each SEC run.

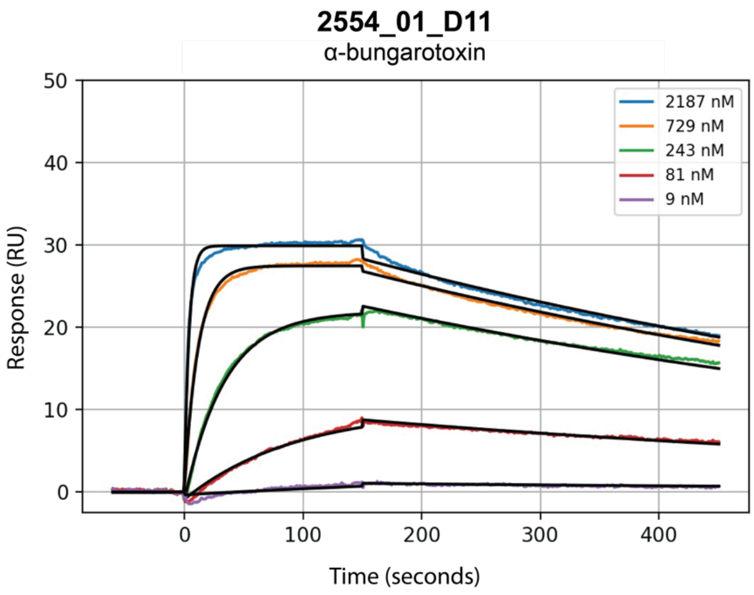

Fig S4. Affinity measurements using surface plasmon resonance. Sensorgram illustrating affinity measurements of D11 on α-bungarotoxin immobilized on a CM5 sensor.
