# Supplementary material for: Discovery and optimization of a broadly-neutralizing human monoclonal antibody against long-chain α-neurotoxins from snakes
Source: Nat Commun. 2023 Feb 8;14:682. doi: 10.1038/s41467-023-36393-4 (PMC9908967; doi:10.1038/s41467-023-36393-4)
Supplement: Supplementary file 2 — Reporting Summary [file 41467_2023_36393_MOESM2_ESM.pdf]

nature portfolio

Corresponding author(s): Andreas Laustsen
Last updated by author(s): 16.12.2022

Reporting Summary

Nature Portfolio wishes to improve the reproducibility of the work that we publish. This form provides structure for consistency and transparency in reporting. For further information on Nature Portfolio policies, see our Editorial Policies and the Editorial Policy Checklist.

Statistics

For all statistical analyses, confirm that the following items are present in the figure legend, table legend, main text, or Methods section.

n/a	Confirmed	
☒	☐	The exact sample size (*n*) for each experimental group/condition, given as a discrete number and unit of measurement
☒	☐	A statement on whether measurements were taken from distinct samples or whether the same sample was measured repeatedly
☒	☐	The statistical test(s) used AND whether they are one- or two-sided<br>*Only common tests should be described solely by name; describe more complex techniques in the Methods section.*
☒	☐	A description of all covariates tested
☒	☐	A description of any assumptions or corrections, such as tests of normality and adjustment for multiple comparisons
☐	☒	A full description of the statistical parameters including central tendency (e.g. means) or other basic estimates (e.g. regression coefficient) AND variation (e.g. standard deviation) or associated estimates of uncertainty (e.g. confidence intervals)
☒	☐	For null hypothesis testing, the test statistic (e.g. *F*, *t*, *r*) with confidence intervals, effect sizes, degrees of freedom and *P* value noted<br>*Give P values as exact values whenever suitable.*
☒	☐	For Bayesian analysis, information on the choice of priors and Markov chain Monte Carlo settings
☒	☐	For hierarchical and complex designs, identification of the appropriate level for tests and full reporting of outcomes
☒	☐	Estimates of effect sizes (e.g. Cohen's *d*, Pearson's *r*), indicating how they were calculated

Our web collection on statistics for biologists contains articles on many of the points above.

Software and code

Policy information about availability of computer code

Data collection	NA
Data analysis	Peptide sequence assignment was performed for 1+ fragmentation ions using the BioLynx package, which is a part of the MassLynx v4.1 software. Sequence alignment was performed in Clustal Omega 1.2.2 and visualized in Jalview 2.11.2.0. Structural alignment and root-mean-square deviation (RMSD) analysis were performed in ChimeraX 1.3. Epitopes of P01388 and P01396 were identified using the STAB Profiles tool 1.0. Kaplan-Meier plots generated with Prism v.9.4.1.

For manuscripts utilizing custom algorithms or software that are central to the research but not yet described in published literature, software must be made available to editors and reviewers. We strongly encourage code deposition in a community repository (e.g. GitHub). See the Nature Portfolio guidelines for submitting code & software for further information.

Data

Policy information about availability of data

All manuscripts must include a data availability statement. This statement should provide the following information, where applicable:

- Accession codes, unique identifiers, or web links for publicly available datasets
- A description of any restrictions on data availability
- For clinical datasets or third party data, please ensure that the statement adheres to our policy

The data that supports the findings of this study are available from the corresponding authors upon reasonable request.

Human research participants

Policy information about studies involving human research participants and Sex and Gender in Research.

Reporting on sex and gender	NA
Population characteristics	NA
Recruitment	NA
Ethics oversight	NA

Note that full information on the approval of the study protocol must also be provided in the manuscript.

Field-specific reporting

Please select the one below that is the best fit for your research. If you are not sure, read the appropriate sections before making your selection.

[x] Life sciences [ ] Behavioural & social sciences [ ] Ecological, evolutionary & environmental sciences

For a reference copy of the document with all sections, see nature.com/documents/nr-reporting-summary-flat.pdf

Life sciences study design

All studies must disclose on these points even when the disclosure is negative.

Sample size	Sample sizes (in particular in the rodent models employed) were chosen based on standard sample sizes involved in venom neutralisation studies with cohorts of 4 animals.
Data exclusions	No data was excluded
Replication	All replicates were successful
Randomization	Not relevant in lethality studies involving venom neutralization, as the outcome (death) is clearly evident
Blinding	Blinding is not relevant in lethality studies involving venom neutralization, as the outcome (death) is clearly evident

Reporting for specific materials, systems and methods

We require information from authors about some types of materials, experimental systems and methods used in many studies. Here, indicate whether each material, system or method listed is relevant to your study. If you are not sure if a list item applies to your research, read the appropriate section before selecting a response.

Materials & experimental systems

n/a	Involved in the study	
[ ]	[x]	Antibodies
[ ]	[x]	Eukaryotic cell lines
[x]	[ ]	Palaeontology and archaeology
[ ]	[x]	Animals and other organisms
[x]	[ ]	Clinical data
[x]	[ ]	Dual use research of concern

Methods

n/a	Involved in the study	
[x]	[ ]	ChIP-seq
[x]	[ ]	Flow cytometry
[x]	[ ]	MRI-based neuroimaging

Antibodies

Antibodies used	The IONTAS Antibody Library is proprietary and not available freely,
Validation	From manufacturers websites<br>anti-FLAG M2 antibody (Sigma, F1804)<br>Specificity:Detects a single band of protein on a Western Blot from mammalian crude cell lysates by chemiluminescent probing<br>Sensitivity Test Conforms Detects 2 ng of FLAG-BAP fusion protein by Dot Blot using Chemiluminescent Detection.<br>anti-human antibody conjugated with Europium (Perkin Eimers, 1244-330)

DELFIA'' Eu-Labeled Anti-Human lgG is intended for use as a secondary antibody in various types of immunoassay based on solid-phase separation. The label, Eu3+, is measured using dissociation-enhanced time-resolved fluorometry.
The products are made from in vitro produced and affinity-purified mouse monoclonal lgG2b antibody specific for the Fe-part of human lgG. The antibodies recognize all lgG subclasses and cross-react with rabbit immunoglobulins.
This product is supplied ready-to-use in 50 mmol/L Tris-HCI buffered saline solution. As a general guideline for DELFIA'' immunoassays, a final concentration of 100 ng/ml of the anti-human lgG antibodies may be suitable for generation of the signal.

Eukaryotic cell lines

Policy information about cell lines and Sex and Gender in Research

Cell line source(s)	Gibco® CHO_S™ Cells: Gibco® CHO_S™ Cells (cGMP Banked) and Media Kit have been developed for the growth of Chinese Hamster Ovary (CHO) cells and expression of recombinant proteins in suspension culture. Parental CHO-S™ cells have been produced, banked and tested to meet current Good Manufacturing Practice regulations 21 CFR Parts 210, 211, 600 and 610. The cells have been adapted to CD CHO Medium for serum free suspension growth. The CD CHO Medium is made from animal origin free and chemically defined components. It contains no proteins, hydrolysates, or components of unknown composition. The medium is formulated without L-glutamine for greater stability, and without phenol red to minimize potential for estrogen-like effects.
Authentication	yes: cGMP Banked
Mycoplasma contamination	The cells are tested negative for mycoplasma contamination: cGMP Banked
Commonly misidentified lines (See ICLAC register)	NA

Animals and other research organisms

Policy information about studies involving animals; ARRIVE guidelines recommended for reporting animal research, and Sex and Gender in Research

Laboratory animals	Animal experiments were conducted in CD-1 mice of both sexes weighing 18-20 g (corresponding to 4-5 weeks old). Mice were supplied by Instituto Clodomiro Picado and experiments were conducted following protocols approved by the Institutional Committee for the Use and Care of Animals (CICUA), University of Costa Rica (approval number CICUA 82-08). Mice were provided food and water ad libitum and housed in Tecniplast Eurostandard Type II 1264C cages (L25.0 cm x W40.0 cm x H14.0 cm) in groups of 4 mice per cage. Animals were maintained at 18-24 °C, 60-65% relative humidity and 12:12 light-dark cycle.
Wild animals	NA
Reporting on sex	Both sexes weighing 18-20 g
Field-collected samples	NA
Ethics oversight	Protocols approved by the Institutional Committee for the Use and Care of Animals (CICUA), University of Costa Rica (approval number CICUA 82-08).

Note that full information on the approval of the study protocol must also be provided in the manuscript.
